# Astrocyte Unfolded Protein Response Induces a Specific Reactivity State that Causes Non-Cell-Autonomous Neuronal Degeneration

**DOI:** 10.1016/j.neuron.2019.12.014

**Published:** 2020-03-04

**Authors:** Heather L. Smith, Oliver J. Freeman, Adrian J. Butcher, Staffan Holmqvist, Ibrahim Humoud, Tobias Schätzl, Daniel T. Hughes, Nicholas C. Verity, Dean P. Swinden, Joseph Hayes, Lis de Weerd, David H. Rowitch, Robin J.M. Franklin, Giovanna R. Mallucci

**Affiliations:** 1 Department of Clinical Neurosciences and UK Dementia Research Institute at the University of Cambridge, Island Research Building, Cambridge Biomedical Campus, Cambridge, UK; 2 Wellcome-MRC Cambridge Stem Cell Institute, Jeffrey Cheah Biomedical Centre, Cambridge Biomedical Campus, Cambridge, UK; 3 MRC Toxicology Unit, Hodgkin Building, Leicester, UK

**Keywords:** astrocytes, astrocyte reactivity state, neurodegeneration, synapse, secretome, neuroprotection, unfolded protein response, PERK signalling, LCN2, translational neuroscience

## Abstract

Recent interest in astrocyte activation states has raised the fundamental question of how these cells, normally essential for synapse and neuronal maintenance, become pathogenic. Here, we show that activation of the unfolded protein response (UPR), specifically phosphorylated protein kinase R-like endoplasmic reticulum (ER) kinase (PERK-P) signaling—a pathway that is widely dysregulated in neurodegenerative diseases—generates a distinct reactivity state in astrocytes that alters the astrocytic secretome, leading to loss of synaptogenic function *in vitro*. Further, we establish that the same PERK-P-dependent astrocyte reactivity state is harmful to neurons *in vivo* in mice with prion neurodegeneration. Critically, targeting this signaling exclusively in astrocytes during prion disease is alone sufficient to prevent neuronal loss and significantly prolongs survival. Thus, the astrocyte reactivity state resulting from UPR over-activation is a distinct pathogenic mechanism that can by itself be effectively targeted for neuroprotection.

## Highlights

• PERK-eIF2α signaling in astrocytes generates a distinct “UPR”-reactivity state
• UPR-reactive astrocytes have an altered secretome, with reduced synaptogenic factors
• UPR-reactive astrocytes fail to support synaptogenesis *in vitro*
• Targeting the astrocytic UPR prevents synapse and neuronal loss in prion-diseased mice

## Graphical Abstract

**Figure:**
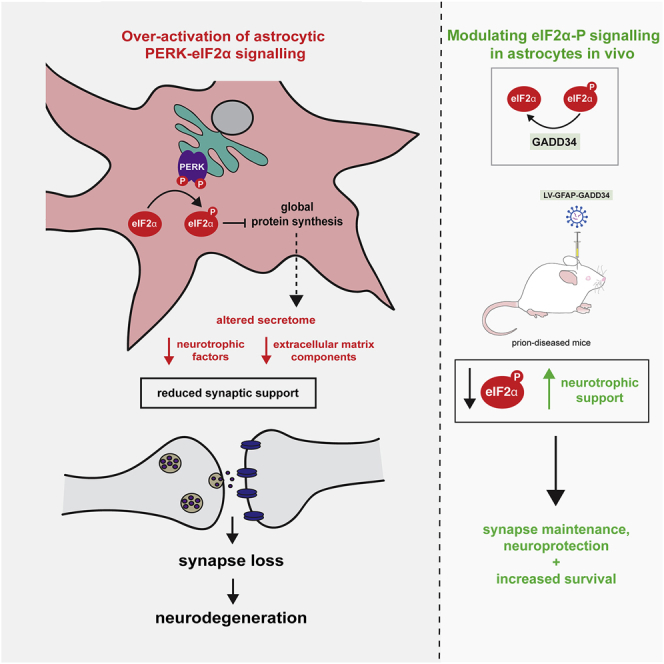

## Teaser

Dysregulation of UPR signaling in neurons is a key mediator of neurodegeneration. Smith et al. show that UPR dysregulation in astrocytes impairs their ability to support synapses; modulating astrocytic UPR signaling prevents neuronal loss and increases survival in prion-diseased mice.

## Introduction

**Figure 1:**
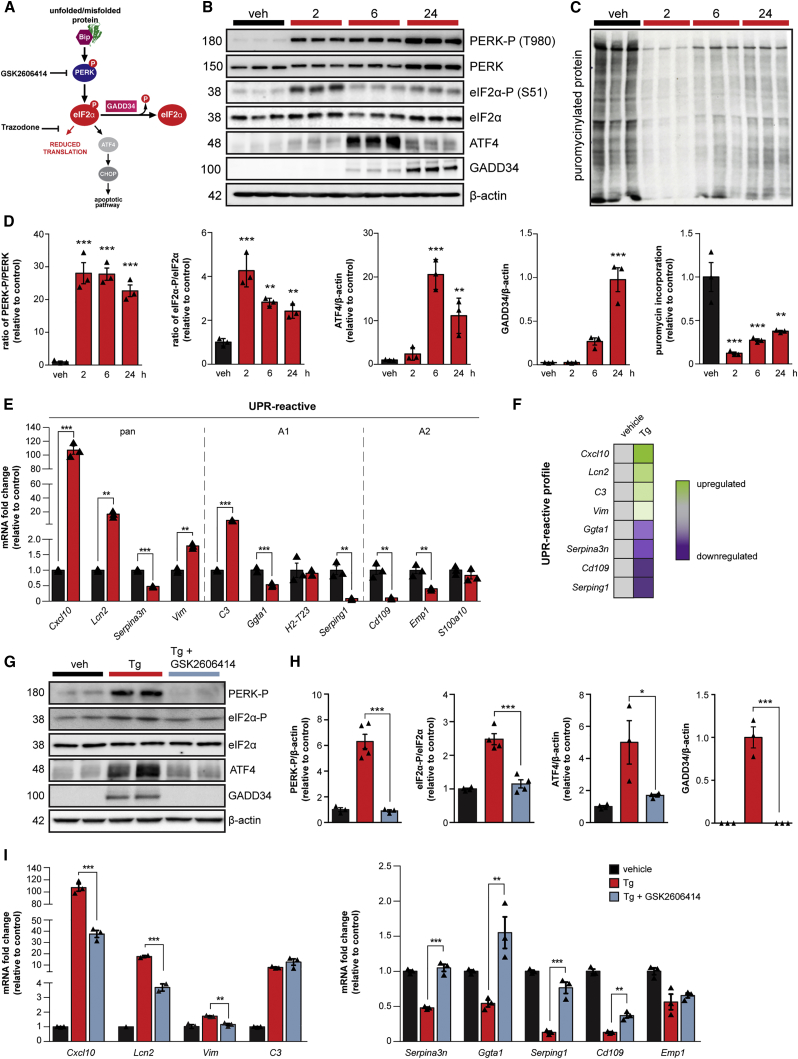
PERK-eIF2α Signaling Induces a Reactive Phenotype in Primary Cultured Astrocytes (A) Schematic of the PERK branch of the UPR, including the sites of action of the PERK branch inhibitors, GSK2606414 and trazodone. (B) Western blots showing PERK-eIF2α signaling in primary astrocytes treated with 300 nM thapsigargin (Tg) for 2, 6, or 24 h. (C) Primary astrocytes show reduced protein synthesis rates in the presence of Tg, as measured by the incorporation of puromycin into nascent proteins. (D) Quantification of western blots. (E) qPCR analysis of astrocyte reactivity markers revealed an altered profile on Tg treatment. (F) Astrocyte reactivity markers that characterize the UPR-reactive profile. (G) PERK-eIF2α signaling is significantly reduced in primary astrocytes cultured in the presence of Tg and 5 μM GSK2606414. (H) Western blots quantified. (I) GSK2606414 significantly blunts the reactivity profile of Tg-stressed astrocytes. All bar graphs show mean ± SEM. ^∗^p < 0.05; ^∗∗^p < 0.01; ^∗∗∗^p < 0.001; one-way ANOVA. n = 3 biological replicates.

The buildup of misfolded proteins, characteristic of many neurodegenerative diseases, is a cellular stress that results in activation of the unfolded protein response (UPR), a signaling cascade that aims to restore protein homeostasis (Ron and Walter, 2007). One branch of the UPR, mediated by phosphorylation of protein kinase R-like ER kinase (PERK), leads to the transient shutdown of protein synthesis through phosphorylation of the α subunit of eukaryotic initiation factor 2 (eIF2α) (Figure 1A). In several mouse models of neurodegeneration, chronic PERK-eIF2α-P signaling results in the sustained reduction in global protein synthesis rates, leading to synaptic failure and neuronal loss (Moreno et al., 2012, Radford et al., 2015). Restoration of translation rates in neurons through genetic or pharmacological modulation of PERK-eIF2α-P signaling restores memory and synapse function (Costa-Mattioli et al., 2007, Ma et al., 2013, Sidrauski et al., 2013) and is profoundly neuroprotective in these models (Celardo et al., 2016, Grande et al., 2018, Halliday et al., 2015, Halliday et al., 2017, Kim et al., 2014, Mercado et al., 2018, Moreno et al., 2012, Moreno et al., 2013, Radford et al., 2015). The pathway has become an area of intense interest for the treatment of human neurodegenerative disorders, where increased levels of PERK-P and eIF2α-P occur in association with misfolded protein accumulation in Alzheimer’s and Parkinson’s diseases and related disorders (Hoozemans et al., 2005, Hoozemans et al., 2007, Hoozemans et al., 2009, Smith and Mallucci, 2016, Stutzbach et al., 2013).

Although the neuronal, cell-autonomous effects of dysregulated PERK signaling in neurodegeneration are relatively well understood, the role of astrocytic UPR activation in disease is unknown. Astrocytes are key modulators of synaptic structure and function (Allen et al., 2012, Christopherson et al., 2005, Chung et al., 2013, Kucukdereli et al., 2011), and their activation to a “reactive” state is a common feature of neurodegenerative disorders. Recent observations describe “neurotoxic” and “neuroprotective” reactivity states (Liddelow et al., 2017, Zamanian et al., 2012). However, the mechanisms driving astrocyte reactivity during disease and how this impacts on function and pathogenesis remain unclear. In this study, we show that chronic PERK-eIF2α signaling in astrocytes drives a distinct pathogenic reactivity state both *in vitro* and *in vivo*. This state, which we term “UPR” reactive, is characterized by an astrocytic secretome devoid of synaptogenic properties. It is reversed by PERK inhibition, which restores synaptogenesis *in vitro*. Critically, *in vivo*, modulation of astrocytic PERK-eIF2α signaling in mice with prion disease is profoundly neuroprotective, abrogating clinical signs and prolonging survival, despite ongoing neuronal PERK activation. The pathogenic role of PERK over-activation therefore has a significant, non-cell-autonomous component, generating a distinct reactivity state in astrocytes principally through loss of synaptotrophic function; this provides multiple new targets for restoring synapses and neuroprotection in neurodegenerative diseases with PERK pathway dysregulation.

## Results

### Chronic PERK Branch Activation Drives a Distinct UPR-Reactivity Profile in Primary Cultured Astrocytes

We first confirmed PERK-eIF2α signaling (Figure 1A) in astrocytes *in vitro* and characterized its temporal profile. Primary astrocytes, cultured from wild-type mice, were treated with the ER stressors thapsigargin (Tg) or tunicamycin (Tm) for 2, 6, or 24 h. PERK activation occurred following 2 h of Tg treatment, with significantly increased levels of phosphorylated PERK (PERK-P) and phosphorylated eIF2α (eIF2α-P) at this time point (Figure 1B), consistent with previous reports (Guthrie et al., 2016, Sprenkle et al., 2019). This corresponded with an 88% reduction in global protein synthesis rates at 2 h as a result of rising eIF2α-P levels (Figure 1C). Elevated levels of the downstream markers of eIF2α-P signaling, ATF4 and GADD34, were found after 6 and 24 h of Tg treatment, respectively (Figure 1B). Tm, which activates the UPR through a different mechanism to Tg, drove a similar temporal pattern of PERK-eIF2α signaling (Figures S1B–S1E). Thus, primary cultured astrocytes exhibit a typical PERK-pathway response to ER stressors.

Next, we asked whether PERK signaling affects astrocyte reactivity *in vitro*. We assessed the expression levels of a selection of astrocyte reactivity markers, including “pan,” “A1,” and “A2” markers (Liddelow et al., 2017, Zamanian et al., 2012), following Tg treatment. The A1 reactivity state, described in response to a peripheral inflammatory stimulus, is proposed to be neurotoxic *in vitro*, whereas the A2 reactivity state, described in response to ischemia, is proposed to be neuroprotective (Liddelow et al., 2017, Zamanian et al., 2012). By 24 h of Tg treatment, the pan-reactive markers *Cxcl10*, *Lcn2*, and *Vim* and the A1 marker *C3* were significantly elevated, with 107-fold, 17-fold, 1.8-fold, and 8-fold increases in mRNA levels, respectively (Figure 1E). In contrast, *Ggta1* and *Serping1* (also A1 markers) were significantly reduced, as were *Cd109*, *Emp1* (A2 markers), and the pan-reactive marker, *Serpina3n*, with 47%, 81%, 90%, 60%, and 52% reductions in transcript levels, respectively (Figure 1E). Tm treatment induced a similar reactivity profile at 24 h (Figure S1H). Only *Emp1* and *S100a10* transcript levels differed in the profiles generated by the two ER stressors. Critically, the small molecule GSK2606414, a potent inhibitor of PERK kinase signaling (Axten et al., 2012), prevented the Tg-induced rise in eIF2α-P, ATF4, and GADD34 (Figures 1G and 1H) and, in parallel, significantly reduced the changes to the reactivity profile when co-administered with Tg (Figure 1I) or Tm (Figure S1H), blunting the increase in *Cxcl10*, *Lcn2*, and *Vim*. Trazodone, a drug that acts downstream of eIF2α-P to reverse PERK-eIF2α-P signaling (Halliday et al., 2017), also reduced the elevated expression of *Cxcl10*, *Lcn2*, and *Vim*, as well as *C3*, on Tg treatment (Figure S1I). To exclude any off-target effects of GSK2606414 (Mahameed et al., 2019, Rojas-Rivera et al., 2017), we genetically modulated PERK-eIF2α signaling using small interfering RNA (siRNA) targeting PERK expression. A 73% knockdown of PERK expression significantly reduced the levels of eIF2α-P on Tg treatment and, crucially, lowered *C3*, *Cxcl10*, *Lcn2*, and *Vim*, with 61%, 51%, 82%, and 60% reductions in transcript levels, respectively (Figure S2). Thus, PERK signaling is the primary mediator of a distinct astrocytic reactivity profile *in vitro* (Figure 1F). We term these astrocytes UPR reactive.

### UPR-Reactive Astrocytes Fail to Support Synapses *In Vitro* due to an Altered Secretome

**Figure 2:**
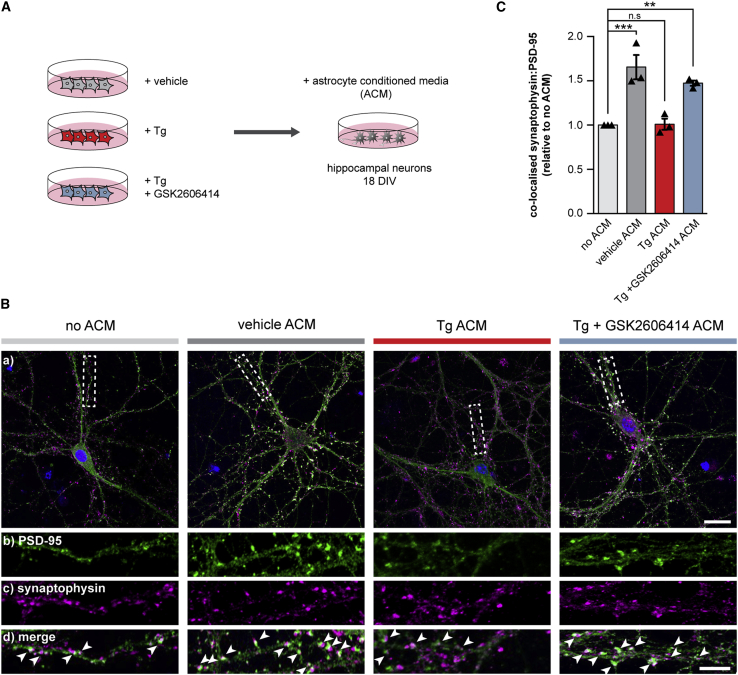
UPR-Reactive Astrocytes Fail to Promote Synaptogenesis *In Vitro* (A) Schematic illustrating the generation of astrocyte-conditioned media (ACM). Primary astrocytes were treated with vehicle, 300 nM Tg, or 300 nM Tg and 5 μM GSK2606414. Astrocytes were washed 24 h post-treatment and incubated with fresh neuron media for a further 24 h. Conditioned media were transferred to primary hippocampal neurons at 18 days *in vitro* (DIV). (B) Representative images of primary hippocampal neurons grown without ACM or with vehicle, Tg, or Tg and GSK2606414 ACM. Neurons were immunostained with the pre-synaptic marker, synaptophysin (magenta) and the post-synaptic marker, PSD-95 (green). Arrows highlight co-localized synaptophysin and PSD-95 puncta. Scale bars, (a) 25 μm; (b–d) 10 μm. (C) Relative number of synapses normalized to the no ACM condition. Tg ACM failed to promote synaptogenesis, whereas Tg and GSK2606414 ACM retained its synaptogenic properties. 10 neurons were counted per condition, per biological replicate (n = 3). Bar graph shows mean normalized synapse number ± SEM. ^∗∗^p < 0.01; ^∗∗∗^p < 0.001; n.s., non-significant; one-way ANOVA.

We next tested for functional effects of the PERK-mediated changes to the astrocyte reactivity state. Astrocytes and astrocyte-conditioned media (ACM) have marked trophic effects on synaptogenesis in primary neuronal cultures (Allen et al., 2012, Christopherson et al., 2005, Ullian et al., 2001). We therefore assessed the synaptogenic properties of conditioned media from UPR-reactive astrocytes (Figure 2A). ACM from vehicle-treated astrocytes significantly increased the synaptic density of primary hippocampal neurons by 1.65-fold (Figures 2B and 2C), consistent with previous reports (Allen et al., 2012). In contrast, ACM from Tg-treated astrocytes had no effect on synapse number (Figures 2B and 2C). PERK inhibition restored the synaptogenic properties of the ACM, with ACM derived from GSK2606414-treated Tg-stressed astrocytes significantly increasing synapse number by 1.47-fold (Figures 2B and 2C). Thus, PERK activation reduces the synaptogenic function of astrocytes as a result of the altered UPR-reactivity state.

**Figure 3:**
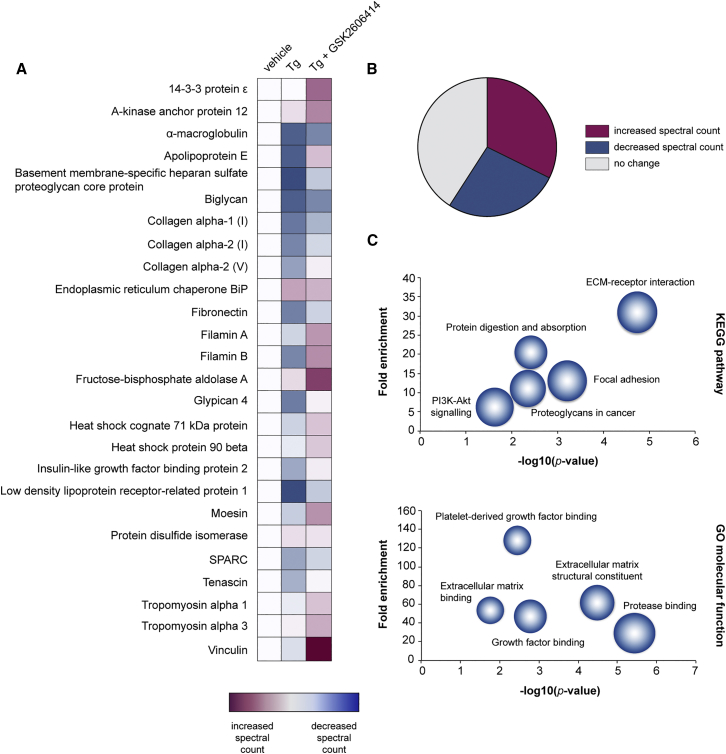
UPR-Reactive Astrocytes Have an Altered Secretome The proteome of conditioned media from vehicle-, Tg-, or Tg and GSK2606414-treated astrocytes was analyzed by LC/MS. (A) Heatmap showing normalized spectral counts of the 25 most abundant proteins detected in the conditioned media of vehicle-treated astrocytes. 10 of the 25 proteins showed reduced spectral counts on Tg treatment. These changes were largely reversed by PERK inhibition. (B) Pie chart showing the percentage of proteins that exhibited an increase, decrease, or no change in spectral counts on Tg treatment. (C) Bubble plots showing KEGG and GO functional analysis of the proteins that displayed reduced spectral counts on Tg treatment, as determined using DAVID. Terms relating to the extracellular matrix and cell adhesion were significantly enriched upon PERK activation (p < 0.0001). Size of bubble represents protein count. LC/MS was performed on conditioned media from 3 biological replicates.

To better understand the mechanisms underlying the loss of astrocytic synaptotrophism upon chronic PERK activation, we compared the secretome of vehicle and Tg-treated astrocytes using unbiased liquid chromatography/mass spectrometry (LC/MS) analysis. Conditioned media from vehicle-treated astrocytes contained an array of proteins essential for synapse maintenance; constituents of the extracellular matrix, such as collagen, fibronectin, and glypican-4, a key synaptogenic factor (Allen et al., 2012), were particularly abundant (Figure 3A; see Table S1 for full list). The abundance of proteins in the secretome was markedly altered by Tg treatment, with 34 out of the 127 proteins detected showing at least a 1.3-fold reduction in spectral counts compared to the secretome of vehicle-treated astrocytes (Figures 3A and 3B). KEGG (Kyoto Encyclopedia of Gene and Genomes) and Gene Ontology analysis revealed that extracellular matrix and cell adhesion pathways were most significantly affected by Tg treatment (p = 6.9 × 10^−7^ and 4.6 × 10^−5^, respectively; Figure 3C). Downregulation of these pathways generally, and of the levels of collagen, fibronectin, and glypican-4, specifically, likely explains the loss of synaptotrophic support from UPR-reactive astrocytes. Interestingly, 41/127 (32%) proteins showed a 1.3-fold or greater increase in spectral counts on Tg treatment (Figure 3B; Table S1). These proteins were largely chaperones involved in protein folding and processing in the ER, consistent with UPR activation, and included BiP and the neurotrophic factor, MANF (Figures 3B and S3; Table S1). 52/127 (41%) proteins showed no change in abundance on Tg treatment (Figure 3B; Table S1). Thus, it is likely that changes to the secretome induced by Tg treatment do not solely reflect the reduction in global protein synthesis rates that accompany PERK activation (Figure 1C) but likely reflect changes in the astrocytic translatome under these conditions. PERK inhibition largely reversed the changes to protein abundance (Figure 3A; Table S1). The UPR-reactivity state resulting from chronic PERK branch activation therefore alters the secretome of primary astrocytes, to the detriment of synaptic trophism.

### Astrocytic PERK-eIF2α Signaling Drives a UPR-Reactivity State *In Vivo* that Is Reversed by Genetic Modulation of the Pathway

Given the effect of PERK signaling on astrocyte reactivity *in vitro* (Figures 1E and S1H), we next examined whether this also occurs *in vivo*. We used the tg37^+/−^ mouse model of prion disease, which has been extensively characterized with respect to PERK-eIF2α signaling and its modulation, particularly in neurons (Halliday et al., 2015, Halliday et al., 2017, Moreno et al., 2012, Moreno et al., 2013). Tg37^+/−^ mice express around ∼3-fold wild-type levels of prion protein (PrP) and succumb to RML (Rocky Mountain Laboratory) prion infection 12 weeks post-inoculation (w.p.i.) (Mallucci et al., 2002, Mallucci et al., 2003, Mallucci et al., 2007). Rising levels of misfolded PrP result in high PERK-P and eIF2α-P and reduced translation rates in the hippocampus from 9 w.p.i., followed by the onset of neuronal loss at 10 w.p.i., with the emergence of overt clinical signs and widespread neurodegeneration by 12 w.p.i. (Moreno et al., 2012). The mice show prominent astrocytosis from 8 w.p.i., with increased GFAP staining over the course of the disease (Figure S4A), consistent with previous reports (Mallucci et al., 2002, Mallucci et al., 2003). PERK-P staining was detected in astrocytes and neurons in the CA1 region of prion-infected mice at 10 w.p.i. (Figure S4B).

**Figure 4:**
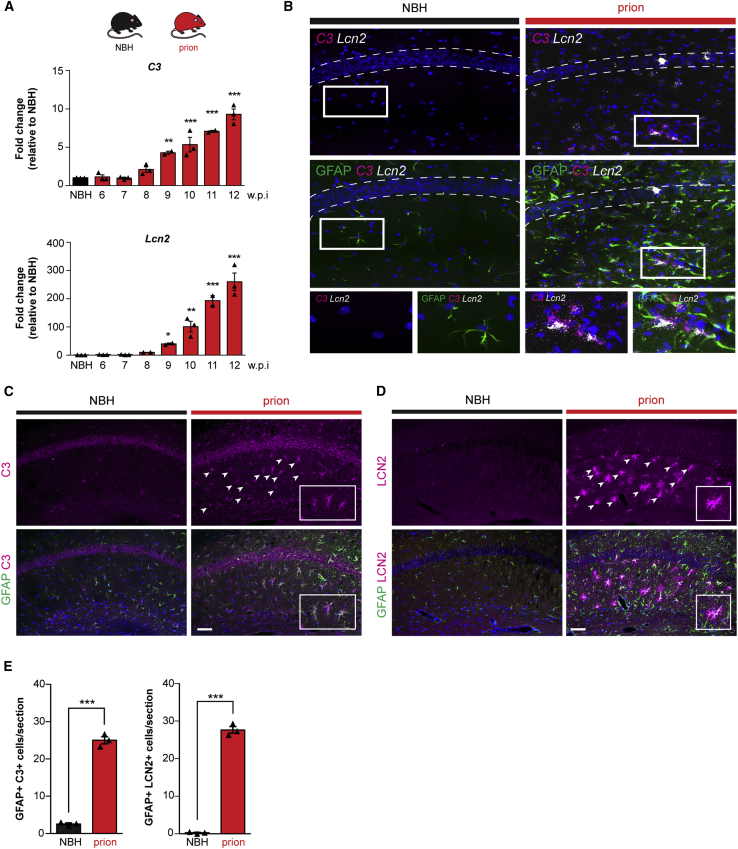
UPR-Reactive Astrocytes Upregulate C3 and LCN2 *In Vivo* (A) qPCR analysis of hippocampal *C3* and *Lcn2* mRNA levels across the prion time course. ^∗^p < 0.05; ^∗∗^p < 0.01; ^∗∗∗^p < 0.001; n = 3 mice per time point. (B) RNA scope showing the localization of *C3* (magenta) and *Lcn2* (white) to GFAP^+^ astrocytes (green) in the hippocampus of prion-diseased mice at 10 w.p.i. (C and D) Immunostaining of C3 (C) and LCN2 (D) in the hippocampus of prion-diseased mice at 10 w.p.i. Scale bars, 100 μm. (E) Prion-diseased mice showed a significant increase in the number of C3^+^/GFAP^+^ and LCN2^+^/GFAP^+^ astrocytes. Bar graphs show mean ± SEM; ^∗∗∗^p < 0.001; Student’s t test. n = 3 mice.

We examined hippocampal homogenates of prion-diseased mice at 10 w.p.i., when PERK signaling is known to be activated (Moreno et al., 2012) and reactive astrocytosis is established (Figure S4A). We tested for expression of UPR-reactivity markers defined *in vitro* (Figures 1E and 1F) and found a pattern of mRNA transcript expression very similar to that seen in Tg- and Tm-stressed astrocytes (Figures 1E, 1F, and S1H). Thus, mRNA levels of *C3*, *Cxcl10*, *Lcn2*, and *Vim* rose significantly from 9 w.p.i. (Figures 4A and S5A), in parallel with increased PERK-eIF2α signaling (Figure S5C), and remained elevated through to the terminal stages of the disease (Figures 4A and S5A). mRNA levels of *Lcn2*, a pan-reactive marker, increased more than 250-fold in terminal prion-diseased mice compared to control tg37^+/−^ mice (Figure 4A). Transcript levels of *Sox9*, an astrocyte-specific nuclear marker, were unchanged through the disease course (Figure S5A), consistent with an altered reactivity state rather than merely reflecting an increase in astrocyte number. RNA scope (Figure 4B) and immunohistochemistry (Figures 4C and 4D) confirmed the astrocytic origins of *C3* and *Lcn2* transcript and protein, respectively, in prion-diseased mice at 10 w.p.i. The rise in these proteins occurred at 9 w.p.i., in line with increased PERK-eIF2α signaling (Figures S5B–S5D).

**Figure 5:**
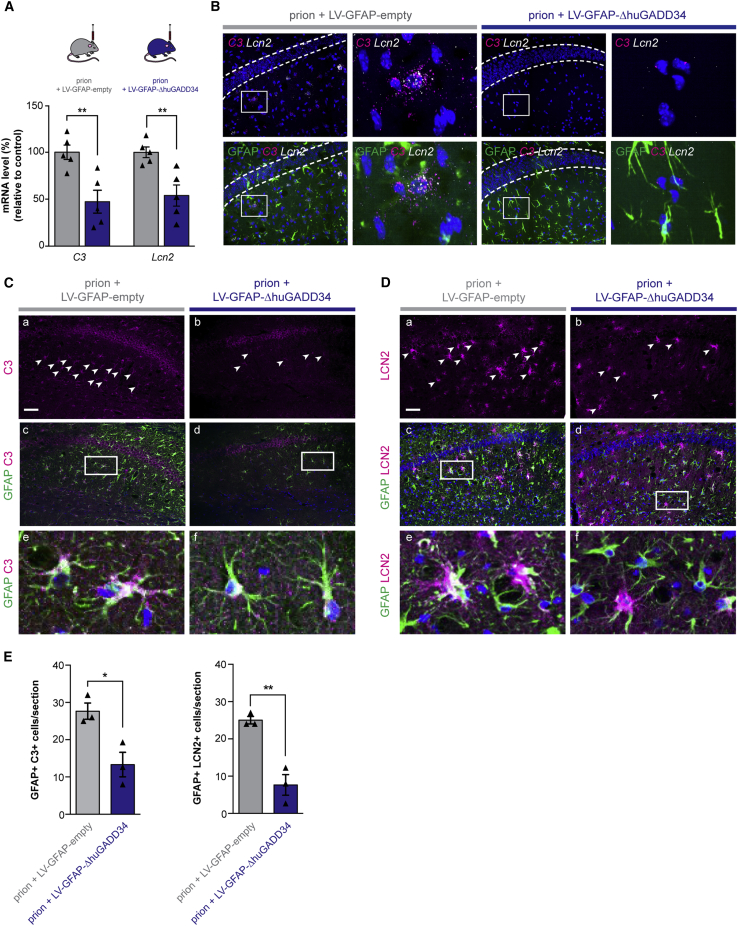
Genetic Modulation of Astrocytic PERK-eIF2α Signaling Ameliorates the UPR-Reactivity State *In Vivo* (A) Prion-inoculated mice were injected with lentivirus at 5 w.p.i., prior to synapse loss. The astrocytic expression of ΔhuGADD34 significantly reduced the mRNA levels of the UPR-reactivity markers *C3* and *Lcn2* at 10 w.p.i., as analyzed by qPCR. ^∗∗^p < 0.01; n = 5 mice per condition. (B) RNA scope also revealed a reduction in *C3* and *Lcn2* upon the expression of ΔhuGADD34. (C and D) Protein levels of C3 (C) and LCN2 (D) were similarly reduced by astrocytic ΔhuGADD34, as determined by immunohistochemistry. Scale bars, 100 μm. (E) Bar graphs represent quantification of C3^+^ and LCN2^+^ astrocytes treated with ΔhuGADD34 or empty virus. Bar graphs show mean ± SEM; ^∗∗∗^p < 0.001; Student’s t test. n = 3 mice.

In order to modulate PERK-eIF2α signaling specifically in astrocytes, we generated lentiviral vectors that overexpress an active fragment of GADD34 (ΔhuGADD34), the specific inducible phosphatase of eIF2α-P, under the astrocytic promoter GFAP (Figure S6A). LV-GFAP-ΔhuGADD34-transduced primary astrocytes (Figure S6B) resulted in reduced eIF2α-P levels and increased protein synthesis rates on Tg treatment (Figures S6C and S6D), confirming both functionality of the virus and efficacy of GADD34 activity in cultured astrocytes. *In vivo*, mice were injected with LV-GFAP-ΔhuGADD34 at 5 w.p.i. to allow for viral expression as disease progressed. Astrocytic expression of the viral construct was confirmed through detection of lentivirally mediated expression of GFP (linked to ΔhuGADD34) in hippocampal sections (Figure S6E). Virally expressed ΔhuGADD34 caused reduced eIF2α-P levels in prion-infected mice at 10 w.p.i. (Figures S6F and S6G). Critically, LV-GFAP-ΔhuGADD34 treatment of prion-infected mice reduced the astrocyte reactivity profile (Figures 5A, S6H, and S6I), consistent with the effects of PERK inhibitor treatment *in vitro* (Figure 1G). Levels of *C3*, *Cxcl10*, *Lcn2*, and *Vim* were reduced by 53%, 63%, 46%, and 35%, respectively, at 10 w.p.i. following LV-GFAP-ΔhuGADD34 injection (Figures 5A and S6I). The reduction in *C3* and *Lcn2* transcript levels in astrocytes of GADD34-treated mice was confirmed by RNA scope (Figure 5B). C3 and LCN2 protein levels were also significantly reduced in LV-GFAP-ΔhuGADD34-treated mice, consistent with reduced astrocyte activation (Figures 5C and 5D).

### Targeting PERK-eIF2α Signaling in Astrocytes *In Vivo* Is Profoundly Neuroprotective

**Figure 6:**
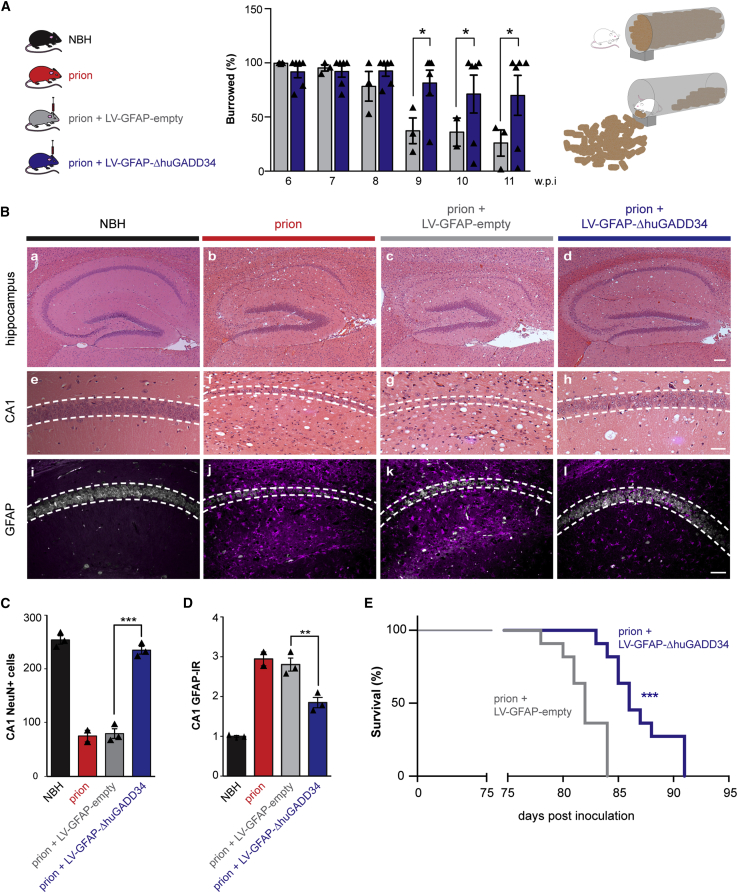
Targeting Astrocytic PERK-eIF2α Signaling Is Neuroprotective in Prion-Diseased Mice (A) Prion-infected mice received hippocampal injections of LV-GFAP-empty or LV-GFAP-ΔhuGADD34 at 5 w.p.i. The overexpression of ΔhuGADD34 prevented the decline in burrowing behavior at 9, 10, and 11 w.p.i. (B) Representative images of hematoxylin-and-eosin-stained hippocampus from normal brain homogenate (NBH) and prion-inoculated mice injected with LV-GFAP-empty or LV-GFAP-ΔhuGADD34. Scale bars, (a–d) 200 μm; (e–j) 50 μm. (C) Astrocytic expression of ΔhuGADD34 resulted in profound neuroprotection. (D) The levels of GFAP were also reduced. (E) Targeting astrocytic PERK-eIF2α significantly prolonged survival. LV-GFAP-empty = 6 mice; LV-GFAP-ΔhuGADD34 = 11 mice. ^∗∗∗^p < 0.001; Mantel-Cox test.

*In vitro*, inhibiting PERK signaling restored the synaptogenic properties of Tg-stressed astrocytes (Figures 2B and 2C). We next asked what were the functional sequelae of modulating PERK signaling in astrocytes *in vivo*, in the context of over-activation of the pathway during prion neurodegeneration. We first observed that lentivirally mediated astrocyte-specific GADD34 expression in prion-diseased mice resulted in a significant and sustained improvement in burrowing behavior (Figure 6A), a motivational task sensitive to hippocampal (notably CA1) integrity that correlates with early synaptic dysfunction in prion disease (Deacon et al., 2001, Mallucci et al., 2007). Control mice treated with an “empty” virus showed a steady decline in burrowing from 8 w.p.i. In parallel, astrocytic expression of GADD34 reduced spongiform degeneration (Figure 6B, h versus f and g) and largely prevented neurodegeneration in the hippocampus (Figure 6B, a–h). Astrocytic GADD34 expression resulted in marked neuroprotection: LV-GFAP-ΔhuGADD34-treated mice showed 67% more pyramidal neurons in the CA1–CA3 region at 12 w.p.i. than mice treated with LV-GFAP-empty or no virus (p = 0.0002; Figures 6B, see h compared to f and g, and 6C). Further, lowering astrocytic PERK signaling through GADD34 overexpression not only reduced the activation state (Figures 5A and S6I) but also significantly decreased astrocyte density (Figures 6B, compare l with j and k, and 6D), with a 35% reduction in astrocyte numbers and markedly less activated morphology in GADD34-treated animals (Figures 5C and 5D, e and f). Finally, lentivirally mediated modulation of astrocytic PERK signaling significantly increased survival in prion-diseased mice to 87 ± 2 days, compared to 82 ± 2 days in mice treated with empty virus (p = 0.0009; Figure 6E). Although this increase may appear modest, this extension of lifespan is comparable to survival data where PERK signaling is similarly modulated in neurons, through lentiviral expression of ΔhuGADD34 under the neuron-specific CAMKII promoter, and is the result of focal modulation only (Figure S6J; Moreno et al., 2012). As with neuronally targeted PERK modulation, levels of PrP and its misfolded disease-associated isoform, PrP^Sc^, were unaffected by the interventions (Figure S6K). These data strongly implicate the role of astrocytic PERK signaling in neuronal demise during disease, through the loss of critical synaptotrophic support.

## Discussion

The processes leading to neurodegeneration involve several cell types other than neurons. The role of microglia in these disorders has been extensively studied, but although astrocyte activation and reactivity are well documented, the mechanisms underlying the astrocytic response to neurodegeneration are still largely unresolved. These cells were the focus of our study.

The pathogenic role of dysregulated PERK-eIF2α-P signaling in neurons has been well characterized across the spectrum of neurodegenerative disorders (Grande et al., 2018, Halliday et al., 2015, Halliday et al., 2017, Hoozemans et al., 2005, Hoozemans et al., 2007, Kim et al., 2014, Mercado et al., 2018, Moreno et al., 2012, Moreno et al., 2013, Radford et al., 2015, Stutzbach et al., 2013). PERK-P has been detected in astrocytes from human tauopathy diseases (Nijholt et al., 2012), but the pathogenic effect of astrocytic PERK signaling, if any, in these disorders is unknown. Interestingly, the integrated stress response (ISR), mediated by eIF2α-P signaling, is dysregulated in astrocytes and oligodendrocytes in a group of neurodegenerative disorders known as vanishing white matter disease (VWMD). In these diseases, mutations in eIF2B, the key interactor of eIF2α-P, lead to demyelination and white matter loss (Schiffmann et al., 1994, van der Knaap et al., 1997) through altered eIF2α-P signaling in these cells. Here, we show that UPR activation in astrocytes, specifically PERK-eIF2α-P signaling, generates a distinct reactivity state both *in vitro* and *in vivo*, associated with loss of astrocytic synaptic trophism and neuroprotective functions. Inhibition of astrocytic PERK signaling reverses this activation state and restores synaptogenic properties to cultured astrocytes (Figures 1I, 2B, and 2C). *In vivo*, it has the dramatic effect of affording extensive, profound neuroprotection with prevention of behavioral deficits and increased survival in prion-diseased mice (Figures 6A–6E). The extended lifespan (Figure 6E) seen with focal hippocampal modulation of astrocytic PERK signaling is consistent with observed protective effects of lentiviral modulation of GADD34 in neurons and other neuroprotective genetic modifications in prion-diseased mice (Moreno et al., 2012, Peretti et al., 2015). This may result from hippocampal neuroprotection modifying behavior in treated mice in a way that allows a modest but significant and consistent increase in survival. Alternatively, it may reflect an extended time course of prion spread when the hippocampus is protected. We found a consistent “UPR-reactive” profile, generated in response to two different ER stressors *in vitro*, Tg and Tm (Figures 1E and S1H), and to the *in vivo* stress of disease-associated protein misfolding (Figures 4A and S5), specifically prion protein PrP^Sc^ here. In each case, the profile is reversed by direct inhibition of PERK-P by GSK2606414 (Figures 1H and S1H) or downstream of PERK, by inhibition of eIF2α-P signaling using trazodone (Figure S1I) or via lentivirally mediated GADD34 expression *in vivo* (Figures 5A and S6I). The UPR-reactive profile shares a number of markers with previously described astrocyte reactivity states. A1 reactivity, which is dependent on microglial activation, in particular, has been suggested as representing a “neurotoxic” phenotype of astrocyte activation and has been attributed to contributing to pathogenesis in a number of human neurodegenerative disorders. In fact, although A1 astrocytes are neurotoxic *in vitro*, the *in vivo* evidence for the A1 state in disease is confined to the presence of elevated C3 expression in *post mortem* samples from a variety of neurodegenerative diseases (Liddelow et al., 2017). C3 elevation forms part of the UPR-reactivity profile, probably because this is a common feature of pathological astrocyte activation. However, the role, if any, of C3 in mediating pathogenesis is unclear, and our data support loss of synaptotrophic function of astrocytes over any direct toxic effect as the central pathological process here. Indeed, recent work identified a very similar profile in prion-diseased wild-type mice to the UPR-reactive state we describe, which the authors termed C3^+^/PrP^Sc^-reactive (Hartmann et al., 2019). Interestingly, the C3^+^/PrP^Sc^-reactivity profile was largely unaffected by eliminating microglia-induced astrocyte activation, through genetic knockout of tumor necrosis factor alpha (TNF-α), interleukin-1α (IL-1α), and C1q, confirming its distinction from the A1 state. Indeed, this state may well respond instead to UPR inhibition, given its similarity to the UPR reactivity we describe in a different prion mouse model. Rather than A1, A2, and “pan” reactivity states, we suggest that there are likely to be multiple reactivity profiles for astrocytes in different diseases, driving pathogenesis through loss of neuronal tropism, with or without a degree of direct toxicity.

The importance of the PERK-mediated reactivity state in astrocytes is apparent through its mechanistic effects of altering the secretome, seen in primary astrocytes (Figure 3). UPR-activated astrocytes display a secretome largely devoid of synaptogenic properties. As a result, the astrocytic component of synapse development in cultured hippocampal neurons is absent (Figure 2B). Many extracellular matrix components, notably collagen, fibronectin, and filamin, known for their importance in synapse formation and maintenance (Ferrer-Ferrer and Dityatev, 2018, Hillen et al., 2018), are reduced in UPR-reactive astrocytes (Figure 3A). PERK inhibition restores this property (Figure 3A). Critically, the secretome changes represent potential targets for synaptic rescue and neuroprotection for treatment of neurodegenerative diseases that would avoid systemic, widespread PERK pathway signaling modulation. Indeed, restoring synapse levels through synaptogenic pathways involving the cold-shock proteins RBM3 and RTN3 reverses cognitive deficits and prevents neurodegeneration in mouse models of Alzheimer’s and prion disease (Bastide et al., 2017, Peretti et al., 2015) and is a powerful neuroprotective strategy. A more in-depth analysis of the astrocytic secretome—and proteome—may reveal further small molecules or peptides with synaptogenic properties that could be candidates for therapeutic intervention.

In conclusion, dysregulated PERK signaling in astrocytes generates a reactivity state that drives a marked non-cell-autonomous mechanism of pathogenesis in prion neurodegeneration, likely to be relevant across the spectrum of these disorders in which PERK activation occurs. These include the tauopathies, including Alzheimer’s disease and many other protein misfolding disorders. Targeting the astrocytic UPR, remarkably, is as protective as targeting the neuronal component. The data reveal new mechanisms by which astrocyte reactivity states contribute to neurodegeneration and provide novel potential synapse-specific targets for therapy.

## STAR★Methods

### Key Resources Table

**Table:** 

REAGENT or RESOURCE	SOURCE	IDENTIFIER
**Antibodies**		

Rabbit anti-PERK-P (T980)	Cell Signaling Technology	Cat# 3179; RRID: AB_2095853
Rabbit anti-PERK	Cell Signaling Technology	Cat# 3192; RRID: AB_2095847
Rabbit anti-eIF2α-P (S51)	Cell Signaling Technology	Cat# 3597; RRID: AB_390740
Mouse anti-eIF2α	Cell Signaling Technology	Cat# 2103; RRID: AB_836874
Rabbit anti-ATF4	Santa Cruz Biotechnology	Cat# sc-390063; RRID: AB_2810998
Rabbit anti-GADD34	Proteintech	Cat# 10449-1-AP; RRID: AB_2168724
Mouse anti-puromycin	Millipore	Cat# MABE343; RRID: AB_2566826
Rabbit anti-β-actin	Abcam	Cat# ab8227; RRID: AB_2305186
Rabbit anti-synaptophysin	Synaptic Systems	Cat# 101 002; RRID: AB_887905
Mouse anti-PSD-95	Millipore	Cat# MABN68; RRID: AB_10807979
Goat anti-C3	R&D Systems	Cat# AF2655; RRID: AB_2066622
Goat anti-LCN2	R&D Systems	Cat# AF1857; RRID: AB_355022
Chicken anti-GFAP	Abcam	Cat# ab4674; RRID: AB_304558
Mouse anti-NeuN	Abcam	Cat# ab104224; RRID: AB_10711040
Rabbit anti-Iba1	Abcam	Cat# ab178846; RRID: AB_2636859
Rabbit anti-PrP	D-GEN	Cat# ICSM35

**Bacterial and Virus Strains**		

LV-CAMKII-GFP	This paper	N/A
LV-CAMKII-ΔhuGADD34	This paper	N/A
LV-GFAP-GFP	This paper	N/A
LV-GFAP-ΔhuGADD34	This paper	N/A

**Biological Samples**		

Mouse brain tissue	Strains listed in this table	N/A

**Chemicals, Peptides, and Recombinant Proteins**		

Thapsigargin	Thermo Fisher Scientific	Cat# T7459; CAS: 67526-95-8
Tunicamycin	Sigma-Aldrich	Cat# T7765; CAS: 11089-65-9
GSK2606414	GlaxoSmithKline	N/A
Trazodone	Sigma-Aldrich	Cat# T6154; CAS: 25332-39-2
TNFα	Cell Signaling	Cat# 8902
IL-1α	Sigma-Aldrich	Cat# I3901
C1q	Biorad	Cat# 2221-5504

**Critical Commercial Assays**		

Ggta1 Taqman assay	Thermo Fisher Scientific	Cat# Mm01333302_m1
H2-T23 Taqman assay	Thermo Fisher Scientific	Cat# Mm00439246_g1
Serping1 Taqman assay	Thermo Fisher Scientific	Cat# Mm00437835_m1
Cd109 Taqman assay	Thermo Fisher Scientific	Cat# Mm00462151_m1
Emp1 Taqman assay	Thermo Fisher Scientific	Cat# Mm00515678_m1
S100a10 Taqman assay	Thermo Fisher Scientific	Cat# Mm00501458_g1

**Experimental Models: Cell Lines**		

Mouse: C57BL/6N primary cortical astrocytes	Generated in house	N/A
Mouse: C56BL/6N primary hippocampal neurons	Generated in house	N/A

**Experimental Models: Organisms/Strains**		

tg37^+/−^ mice	Imperial College, London	Mallucci et al., 2002
C57BL/6N mice	Charles River	Strain code: 027

**Oligonucleotides**		

Oligonucleotides for qPCR (see Table S2 for sequences)	Sigma Aldrich	N/A
esiRNA universal negative control	Sigma-Aldrich	Cat# SIC001
esiRNA targeting mouse Eif2ak3	Sigma-Aldrich	Cat# EMU014751

**Recombinant DNA**		

LV-CAMKII-GFP	Vigene Biosciences	Custom made
LV-CAMKII-ΔhuGADD34	Vigene Biosciences	Custom made
LV-GFAP-GFP	Vigene Biosciences	Custom made
LV-GFAP-ΔhuGADD34	Vigene Biosciences	Custom made

**Software and Algorithms**		

Prism	GraphPad	https://www.graphpad.com
ImageJ	National Institute of Health	https://imagej.nih.gov/ij/docs/guide/user-guide.pdf
Fiji	Fiji	https://fiji.sc
Photoshop CS6	Adobe Inc.	https://www.adobe.com/products/photoshop.html
Illustrator CS6	Adobe Inc.	https://www.adobe.com/products/illustrator.html

### Lead Contact and Materials Availability

Further information and requests for resources and reagents should be directed to and will be fulfilled by the Lead Contact, Giovanna Mallucci (gm522@cam.ac.uk). All unique reagents generated in this study are available from the Lead Contact with a completed Materials Transfer Agreement.

### Experimental Model and Subject Details

#### Animals

Tg37^+/−^ mice, on a FVB background, were housed in a temperature-controlled room and were maintained on a 12:12 light dark cycle, with access to food and water *ad libitum*. Both males and females were used in this study. Mice were inoculated with 1% brain homogenate of Chandler/Rocky Mountain Laboratory (RML) prions or normal brain homogenate (NBH) at 3-4 weeks of age, as previously described (Moreno et al., 2012). Prion-inoculated mice were monitored daily and were culled upon the appearance of confirmatory prion signs, which occur around 12 weeks post inoculation. Mice were randomly assigned to experimental groups. All animal work conformed to UK Home Office regulations and institutional and ARRIVE guidelines.

#### Primary astrocyte culture

Primary astrocytes were isolated from the cortices of both male and female P1 C57BL6/N pups. Briefly, cortical tissue was extracted into Hibernate A media (GIBCO); meninges were removed and tissue was minced using a scalpel blade. The minced tissue was then incubated at 37°C for 5 minutes in fresh Hibernate A media supplemented with 1 mg/mL DNase (Sigma) and 0.25% Trypsin (GIBCO). The suspension was triturated then returned to 37°C for 5 minutes. Trituration was repeated and the suspension was incubated for a further 5 minutes. 10 mL of glial media (MEM, 10% horse serum, 0.6% D-glucose and 1% Penicillin/Streptomycin) was added and trituration was performed. The suspension was strained then centrifuged at 200 x g for 5 minutes. The cell pellet was re-suspended in glial media and seeded into T75 flasks (Corning). Cultures were maintained at 37°C, 5% CO_2_ in glial media containing 1% horse serum. Upon reaching confluency, astrocytes were seeded into 6 well plates (Greiner) at a density of 100,000 cells per well.

#### Primary neuron culture

Primary neurons were isolated from the hippocampi of both male and female P1 C57BL6/N pups. Briefly, hippocampi were extracted into Hibernate A media (GIBCO) and incubated at 37°C with papain solution for 20 minutes. Papain solution was removed and trypsin inhibitor was added for 5 minutes. Hippocampi were then washed 3 times in pre-warmed plating media (Neurobasal A, B27 supplement, Glutamax, Horse serum, 1M HEPES pH 7.5) before being triturated 8-10 times. The suspension was strained and 300,000 cells were seeded onto glass coverslips coated with poly-L-lysine. Media was changed to neuron media (Neurobasal A, B27 supplement, Glutamax, Penicillin/Streptomycin) 4 hours post seeding. Primary neurons were maintained at 37°C, 5% CO_2_. A third of the media was changed for fresh media every 3-4 days.

### Method Details

#### Prion infection of mice

All animal work conformed to UK Home Office regulations and institutional and ARRIVE guidelines. Tg37^+/−^ mice (males and females), aged 3-4 weeks, were inoculated with 1% brain homogenate of Chandler/Rocky Mountain Laboratory (RML) prions or normal brain homogenate (NBH), as previously described (Moreno et al., 2012). Animals were culled when they developed clinical signs of prion disease, which occurs around 12 weeks post inoculation.

#### Lentivirus

Lentiviral plasmids were generated by Vigene Biosciences. The catalytically active fragment of human GADD34 was N-terminally FLAG tagged and placed downstream of either a CAMKII or GFAP promoter. A T2a sequence linked ΔhuGADD34 to GFP. Plasmids were packaged by Vigene Biosciences or in house, through the calcium-phosphate transfection of HEK293T cells. Final titers were 1x10^8^ TU/mL.

#### Stereotaxic injection

Prion-infected mice were stereotaxically injected with lentivirus at 5 w.p.i. under general anesthesia. 5 μL of virus was injected using a Hamilton syringe at the following 4 sites relative to bregma: (−2.0 mm, +2.0 mm, −2.2 mm), (−2.7 mm, +2.0 mm, −2.2 mm), (−2.0 mm, −2.0 mm, −2.2 mm) and (−2.7 mm, −2.0 mm, −2.2 mm).

#### Burrowing assay

Burrowing was performed as previously described (Moreno et al., 2013). Briefly, female mice were individually placed in large plastic cages containing a Perspex tube filled with food pellets. The weight of pellets remaining in the tube after 2 hours was measured and the percentage burrowed was calculated.

#### Immunohistochemistry

For neuronal counts, paraffin-embedded brains were sectioned at 4 μm and were stained with hematoxylin and eosin (H&E) or NeuN (Millipore) as previously described (Moreno et al., 2013). CA1 pyramidal neuronal counts were determined with three serial sections from three separate mice. For immunofluorescence, sections were baked at 55°C for 2 hours before being submerged in 10x Heat Mediated Antigen Retrieval Solution pH 6.0 (Abcam) and left overnight at 60°C. Slides were then washed and blocked for 1 hour in 10% goat serum, 0.1% Triton X-100, 0.3M glycine. Primary antibodies were incubated overnight at 4°C. Following washes, Alexa Fluors (Invitrogen) were applied for 1 hour at room temperature. Slides were mounted with ProLong Diamond Antifade with DAPI (Invitrogen). Images were captured on a Leica 4000B microscope.

#### Fluorescence *in situ* RNA hybridization

Fluorescence *in situ* RNA hybridization (FISH) on brain cryosections was automated on BOND RX robotic stainer (Leica). After manual post-fixation, epitope retrieval and dehydration, cryosections were processed for 3-plex FISH using the RNAScope LS Multiplex Assay (ACD) in combination with immunohistochemistry. Tissues were probed against mRNA transcripts *C3* (1:1), *Lcn2* (1:50) and GFAP protein (1:2000). The assay was performed according to the manufacturer’s instructions. In brief, samples were permeabilised with heat and protease treatment to improve probe penetration and hybridization. For heat treatment, samples were incubated in BOND ER2 buffer (pH 9.0, Leica) at 95°C for 2 minutes. For protease treatment, samples were incubated in ACD protease reagent at 42°C for 10 minutes. Prior to probe hybridization, samples were incubated in hydrogen peroxide for 10 minutes to inactivate endogenous peroxidases and ACD protease. Samples were then incubated in target z-probe mixtures for 2 hours at 42°C. Each slide was flushed three times in order to obtain optimal hybridization to transcripts. Following hybridization, branched DNA amplification trees were built through sequential incubations in AMP1, AMP2 and AMP3 reagents for 15-30 minutes, each at 42°C, with LS Rinse buffer (Leica) high stringency washes in-between incubation steps. After amplification, probe channels were detected sequentially by HRP-TSA labeling. Here, samples were incubated in channel-specific HRP reagents for 15 minutes at 42°C, TSA fluorophores for 30 minutes and HRP blocking reagent for 15 minutes at 42°C. Probes were labeled using Atto 425-Streptavidin (Sigma, 40709, 1:400). Directly following FISH assay, localization of GFAP protein was performed by BOND RX assisted IHC. Samples were incubated with anti-GFAP antibody in blocking solution for 1 hour (Abcam). To develop the antibody signal, samples were incubated in donkey anti-goat HRP (Thermo Fisher Scientific) for 1 hour, TSA biotin (PE) for 10 minutes and streptavidin-conjugated Alexa 700-streptavidin (Sigma) for 30 minutes.

#### Fluorescence *in situ* RNA hybridization - imaging

High-resolution FISH images of tissue sections were acquired on a spinning disk Operetta CLS (Perkin Elmer) in confocal mode using a sCMOS camera and a 40X NA 1.1 automated-water dispensing objective. The machine was equipped with custom-made narrow band filters to allow hex-plex imaging. The field-of-view was 320 × 320 μm and voxel size 0.3 × 0.3 × 1 μm. Each field was imaged as a z stack consisting of 20 to 30 planes with a 1 μm step size. Z-heights of tissue sections were manually determined by imaging DAPI on sample fields prior to tissue-wide scans. Each z-plane was imaged across 6 channels with exposure times between 60 and 120 ms at 80% LED power. The background correction was applied uniformly across samples, according to control tissue probed with negative probes and by omitting primary antibody.

#### Western blotting

Protein samples were isolated from hippocampi or astrocyte cell pellets using RIPA lysis buffer (150 μL NaCl, 50 mM Tris, 0.5% sodium deoxycholate, 0.1% SDS and 1% Triton X-100) supplemented with Phos-STOP and protease inhibitors (Roche). 15 μg of protein was resolved by SDS-PAGE then transferred onto nitrocellulose or PVDF membranes. Membranes were incubated overnight with primary antibodies. Horseradish peroxidase secondary antibodies were applied for 1 hour at room temperature at a 1:10,000 dilution (Goat anti-mouse, goat anti-rabbit (BioRad), donkey anti-goat (Promega)). Secondary antibodies were detected with the enhanced chemiluminescence system (ECL, GE Healthcare). β-actin or GAPDH was used as a loading control and quantitative analysis was performed with ImageJ.

#### Co-culture with astrocyte conditioned media

Astrocytes were seeded into 6 well plates at a density of 100,000 cells per well. At 90% confluency, media was removed and cells were washed 3 times with Neurobasal A (GIBCO). Astrocytes were then cultured in neuron media for 24 hours. Vehicle or drug treatments were added for 24 hours. Media was then removed, astrocytes were washed 3 times with Neurobasal A and fresh neuron media was added. Following 24 hours, astrocyte-conditioned neuron media was collected and centrifuged at 200 x g to remove dead cells. Astrocyte-conditioned media was transferred to hippocampal neurons at 18 DIV.

#### Synapse quantification

Neurons were fixed in 4% paraformaldehyde, 4% sucrose for 20 minutes. Coverslips were washed with PBS then incubated with 0.1% Triton X-100 for 5 minutes, followed by 50 mM NH_4_Cl for 20 minutes. Neurons were then blocked in 10% goat serum for 1 hour at room temperature. Synaptophysin (Synaptic Systems) and PSD-95 (Millipore) antibodies were diluted in blocking solution and were incubated overnight at 4°C. Following washes in PBS, neurons were incubated for 1 hour at room temperature with anti-mouse Alexa Fluor 488 and anti-rabbit Alexa Fluor 568 (Thermo). Coverslips were stained with DAPI prior to mounting. Images were captured using a Leica confocal microscope at 63x magnification. The number of co-localized puncta was quantified using the JACoP plugin in ImageJ. Synapse numbers from 10 neurons were counted per condition per biological replicate.

#### LC/MS analysis of astrocyte-conditioned media

Astrocytes were seeded into 6-well plates at a density of 100,000 cells. At 90% confluency, cells were washed 3 times with Neurobasal A (GIBCO), then cultured overnight in serum-free media (Neurobasal A, Glutamax and Penicillin/Streptomycin). Astrocytes were treated for 24 hours with vehicle, thapsigargin or both thapsigargin and GSK2606414. Media was collected and centrifuged for 5 minutes at 4,000 x g to remove any dead cells. Proteins were precipitated using trichloroacetic acid. Precipitates were washed with acetone, resuspended in SDS-PAGE buffer and separated on a 4%–12% gel. The precipitation procedure was also performed on serum-free media alone, as an additional control. Proteins were analyzed by LC/MS. A protein was identified if it received ≥ 99% confidence with ≥ 3 unique peptides at 95% confidence in the vehicle- or thapsigargin-treated condition.

#### Drug treatments

Astrocytes were treated with 300 nM thapsigargin (Thermo) or 3 μg/mL tunicamycin (Sigma) for the indicated time periods. GSK2606414 and trazodone were added at the same time as the ER stressor, at concentrations of 5 μM and 20 μM, respectively. The ‘A1-cocktail’ was composed of TNFα (30 ng/mL), C1q (400 ng/mL) and IL-1α (3 ng/mL).

#### qPCR

Total RNA was isolated with the RNeasy micro kit (QIAGEN), according to manufacturer’s instructions. mRNA was reverse transcribed using the Superscript IV First-Strand Synthesis system (Thermo) and qPCR was performed with Power SYBR Green master mix (Applied Biosystems). Primers were designed using NCBI primer Basic Local Alignment Search Tool (BLAST) software. Primer sequences are listed in Table S2. Primer pairs spanned exon-exon junctions, to avoid amplification of genomic DNA. 300 nM forward primer, 300 nM reverse primer and 1 μL cDNA was used per reaction. qPCR was performed using the QuantStudio 7 Real-Time System (Applied Biosystems) with the following thermal cycling conditions: 95°C for 10 minutes, followed by 40 cycles of 95°C for 15 s, 60°C for 1 minute. All primers pairs amplified a single product, as determined using a melt curve. Data was processed using QuantStudio 6 and 7 Flex software and is expressed as 2^-ΔCT^.

### Quantification and Statistical Analysis

Statistical details for each experiment, including *n* numbers and the statistical test performed, can be found in the corresponding figure legend. Data are presented as mean ± standard error of the mean (SEM). Statistical analysis was performed using Prism V6 software. Student’s t test was used for datasets with a normal distribution and a single intervention. One-way ANOVA was performed with Tukey’s post hoc test for multiple comparisons. Survival data was analyzed using Mantel-Cox. p < 0.05 was considered statistically significant.

### Data and Code Availability

The published article includes all datasets generated during this study.

## Supplemental Information

Document S1. Figures S1–S6 and Tables S1 and S2

Document S2. Article plus Supplemental Information
