# Supplementary material for: Astrocyte Unfolded Protein Response Induces a Specific Reactivity State that Causes Non-Cell-Autonomous Neuronal Degeneration
Source: Neuron. 2020 Mar 4;105(5):855–866.e5. doi: 10.1016/j.neuron.2019.12.014 (PMC7054837; doi:10.1016/j.neuron.2019.12.014)
Supplement: Document S1. Figures S1–S6 and Tables S1 and S2 [file mmc1.pdf]

Neuron, Volume *105*

Supplemental Information

Astrocyte Unfolded Protein Response Induces a Specific Reactivity State that Causes Non-Cell-Autonomous Neuronal Degeneration

Heather L. Smith, Oliver J. Freeman, Adrian J. Butcher, Staffan Holmqvist, Ibrahim Humoud, Tobias Schätzl, Daniel T. Hughes, Nicholas C. Verity, Dean P. Swinden, Joseph Hayes, Lis de Weerd, David H. Rowitch, Robin J.M. Franklin, and Giovanna R. Mallucci

Figure S1 (related to Figure 1)

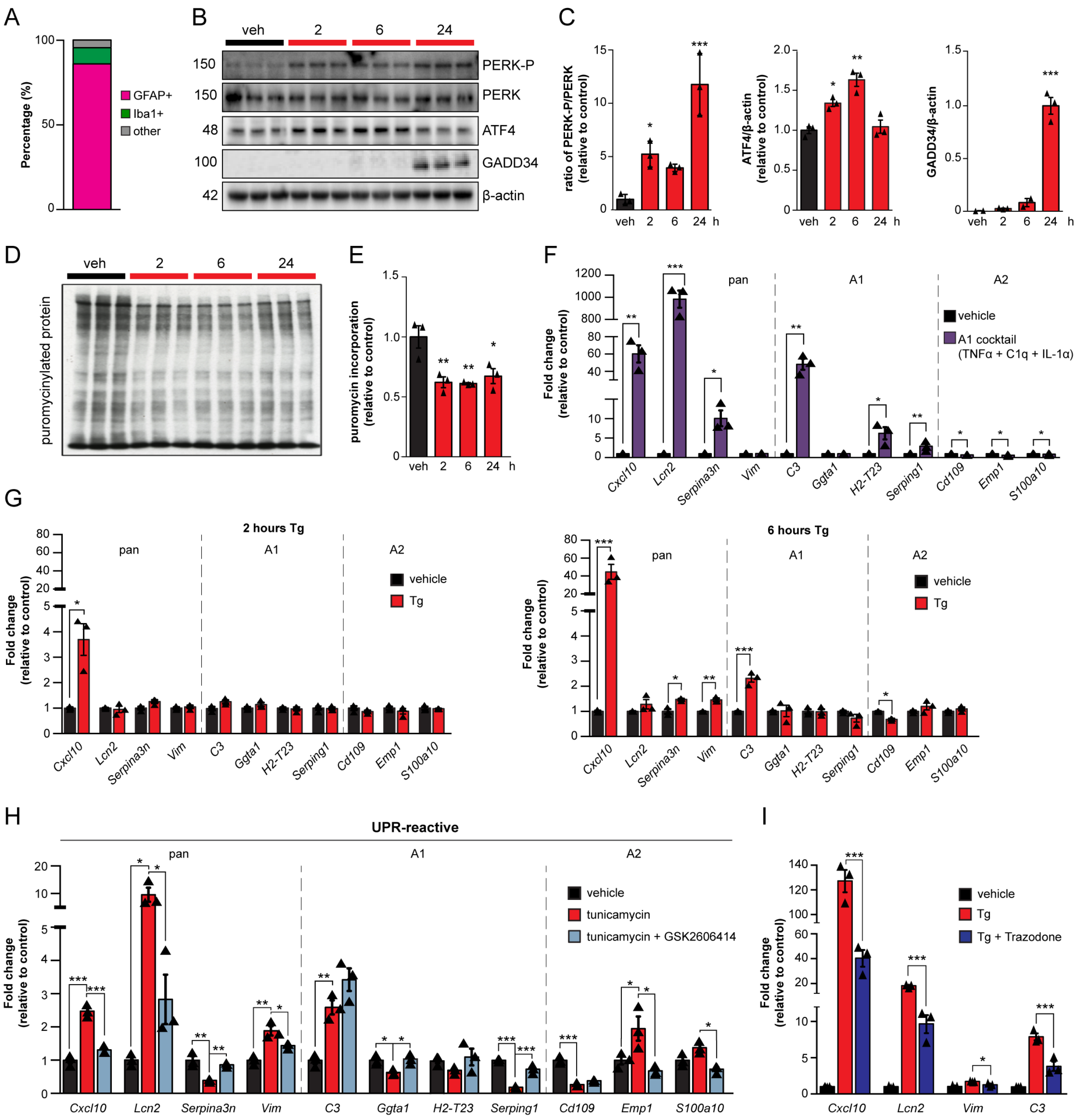

Figure S1 (related to Figure 1). Thapsigargin and tunicamycin induce a reactive phenotype in primary cultured astrocytes. (**A**) Purity of primary astrocyte cultures. Staining for the astrocyte-specific marker, GFAP and the microglial-specific marker, Iba1, revealed a purity of more than 80%. (**B**) Western blot analysis of PERK branch markers in primary astrocytes treated with 3 μg/mL tunicamycin (Tm) for 2, 6 or 24 hours. Quantification of western blots shown in (**C**). (**D**) Protein synthesis rates were reduced following 2 hours of Tm treated, measured using puromycin incorporation into nascent proteins. Quantification of western blot shown in (**E**). (**F**) Positive control for the detection of a 'reactive' astrocyte profile by qPCR. Primary astrocytes were treated with the 'A1-cocktail' (TNFα, C1q and IL-1α) for 24 hours. 'Pan'- and 'A1'-reactive markers were significantly upregulated, whilst 'A2'-reactive markers were downregulated. (**G**) Reactivity profile of primary

astrocytes following 2 or 6 hours of Tg treatment. An altered reactivity state was observed from 6 hours onwards. (**H**) qPCR analysis of reactivity markers following 24 hours of Tm treatment. The PERK inhibitor, GSK2606414, largely restores the reactivity signature observed on ER stress. (**I**) Trazodone (20 µM) partially restores the reactivity profile of Tg-stressed astrocytes, reducing the upregulation of *Cxcl10, Lcn2, Vim* and *C3*. All bar graphs show mean ± SEM. *p <0.05, **p <0.01, ***p <0.001, one-way ANOVA. n = 3 biological replicates.

Figure S2 (related to Figure 1)

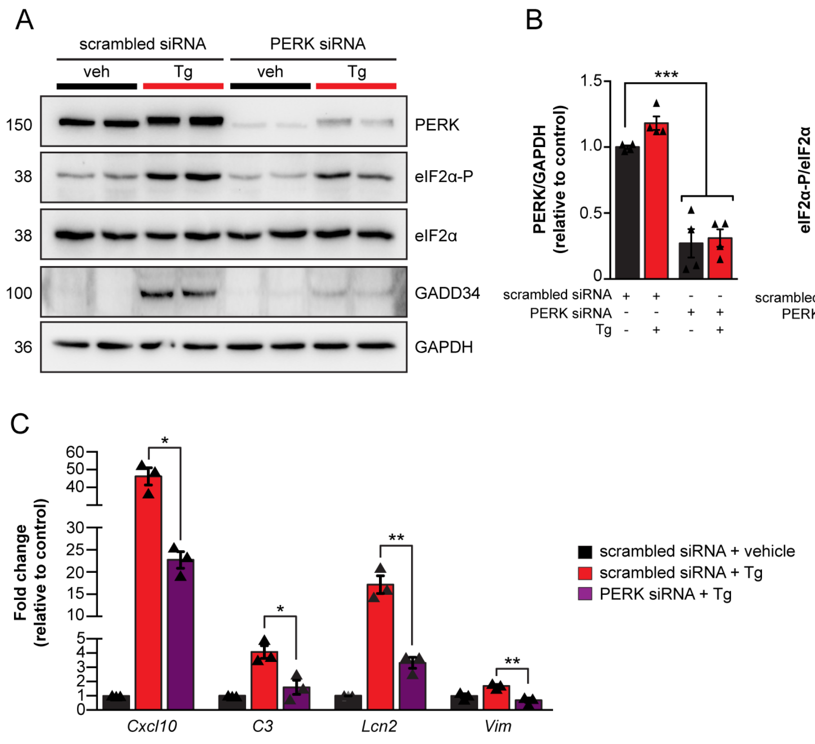

Figure S2 (related to Figure 1). Genetic modulation of PERK-eIF2α signalling reduces astrocyte reactivity on Tg treatment. (**A**) The knockdown of PERK using siRNA significantly lowered the levels of eIF2α-P and GADD34 on Tg treatment. Quantification of western blots shown in (**B**). (**C**) PERK knockdown significantly blunts the upregulation of *Cxcl10, C3, Lcn2* and *Vim* on Tg treatment. All bar graphs show mean ± SEM. *p <0.05, **p <0.01, ***p <0.001, one-way ANOVA. n = 3 biological replicates.

Figure S3 (related to Figure 3)

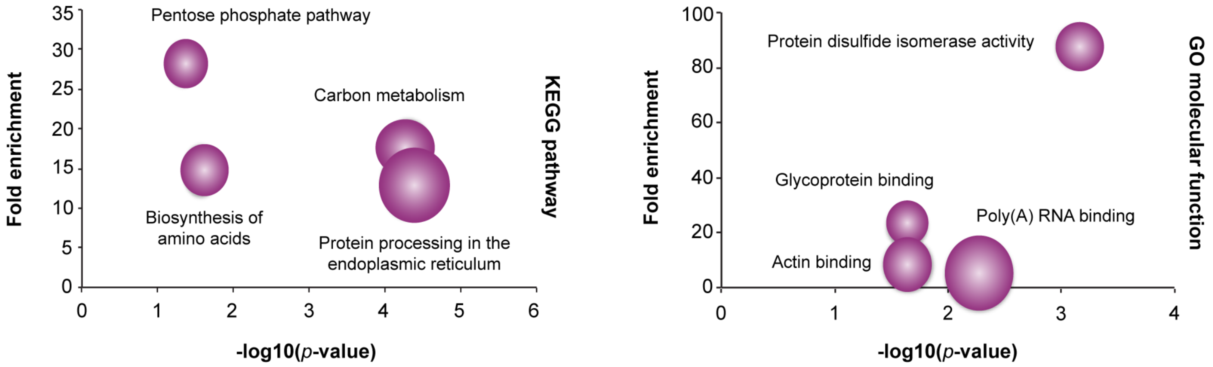

Figure S3 (related to Figure 3). UPR-reactive astrocytes have an altered secretome. Bubble plots showing KEGG and GO functional analysis of proteins that displayed increased spectral counts on Tg treatment, as determined using DAVID. 'Protein processing in the endoplasmic reticulum' and 'protein disulphide isomerase activity' were the most significantly enriched pathways, as determined by KEGG and GO Molecular Function, respectively (*p*-value <0.0001). Size of bubble represents protein count. LC/MS was performed on conditioned media from 3 biological replicates.

Figure S4 (related to Figure 4)

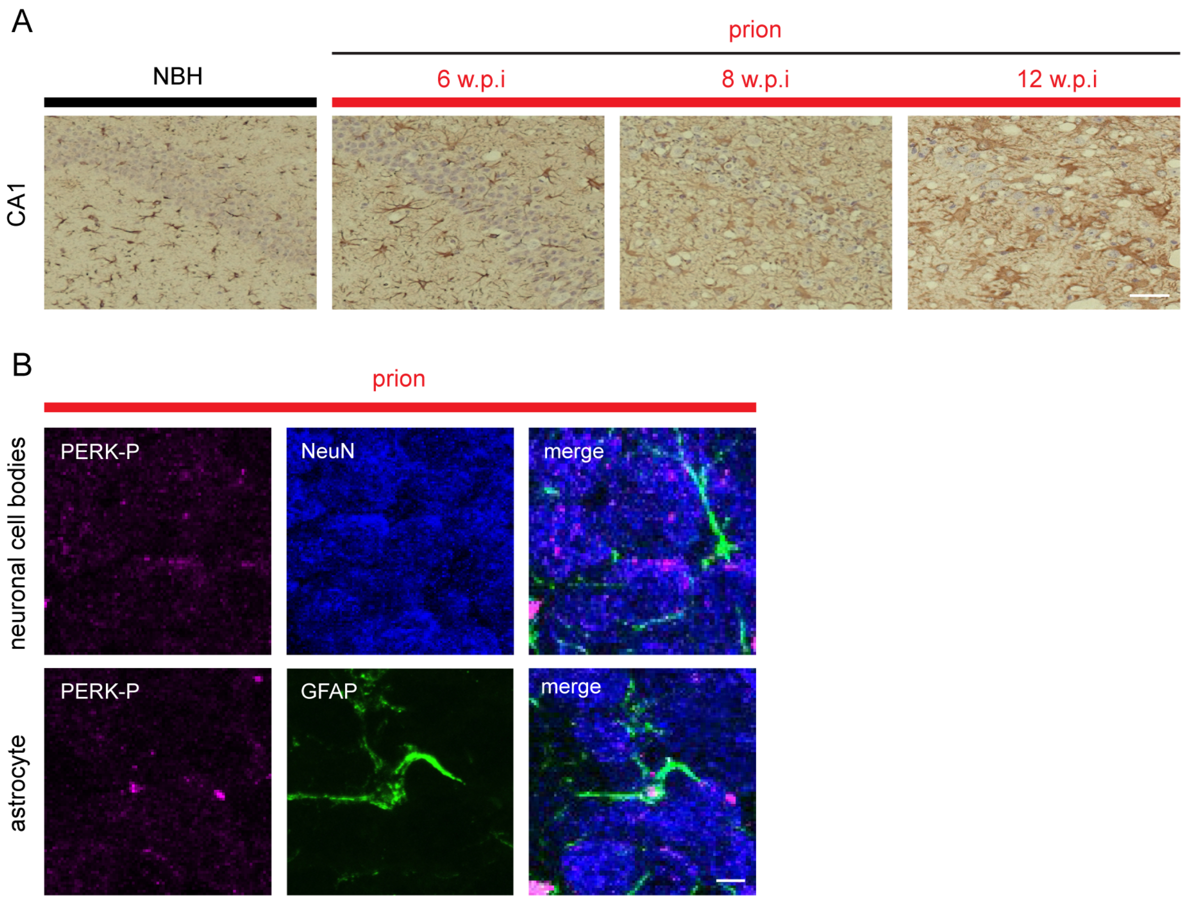

Figure S4 (related to Figure 4). Prion-diseased mice show prominent astrocytosis from 8 w.p.i. (**A**) Representative images of GFAP stained hippocampal sections from NBH and prion-diseased mice illustrating increased astrocytosis across the disease time course. Scale bar, 50 μm (**B**) Hippocampal sections from prion-diseased mice at 10 w.p.i. showing PERK-P staining (magenta) in both the neuronal cell bodies (NeuN, blue) and astrocytes (GFAP, green). Scale bar, 10 μm.

Figure S5 (related to Figure 4)

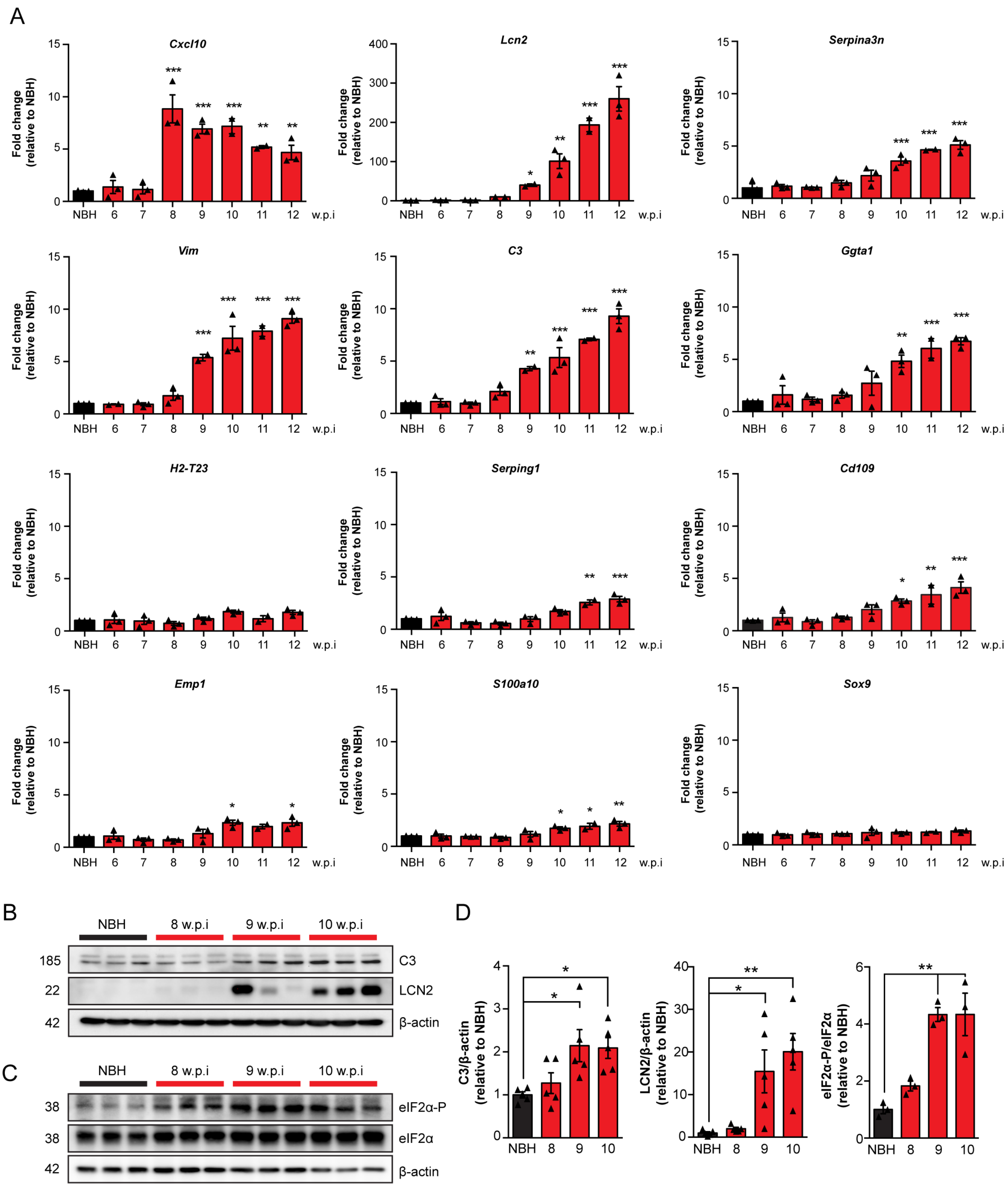

Figure S5 (related to Figure 4). Astrocyte reactivity markers are elevated in prion-diseased mice. (A) qPCR analysis of reactivity markers in hippocampal lysates from NBH and prion-inoculated mice. 9 out of the 11 markers show increased levels from 9 and 10 w.p.i. The upregulation of *Cxcl10* occurs slightly earlier, at 8 w.p.i. The mRNA levels of *Sox9*, a nuclear-specific marker that accounts for astrocyte number, remains unchanged through the course of the disease. Bar graphs show mean ± SEM. **p* <0.05, ***p* <0.01, ****p* <0.001, one-way ANOVA. *n* = 3 mice per time point. (**B**) Western blot analysis of C3 and LCN2 protein levels in hippocampal homogenates from NBH and prion-

diseased mice at 8, 9 and 10 w.p.i. (**C**) The increase in C3 and LCN2 protein at 9 w.p.i. correlates with increased PERK-eIF2α signalling. Quantification of western blots shown in (**D**). Bar graphs show mean ± SEM. *p <0.05, **p <0.01, one-way ANOVA. n = 3-5 mice per time point.

Figure S6 (related to Figures 5 and 6)

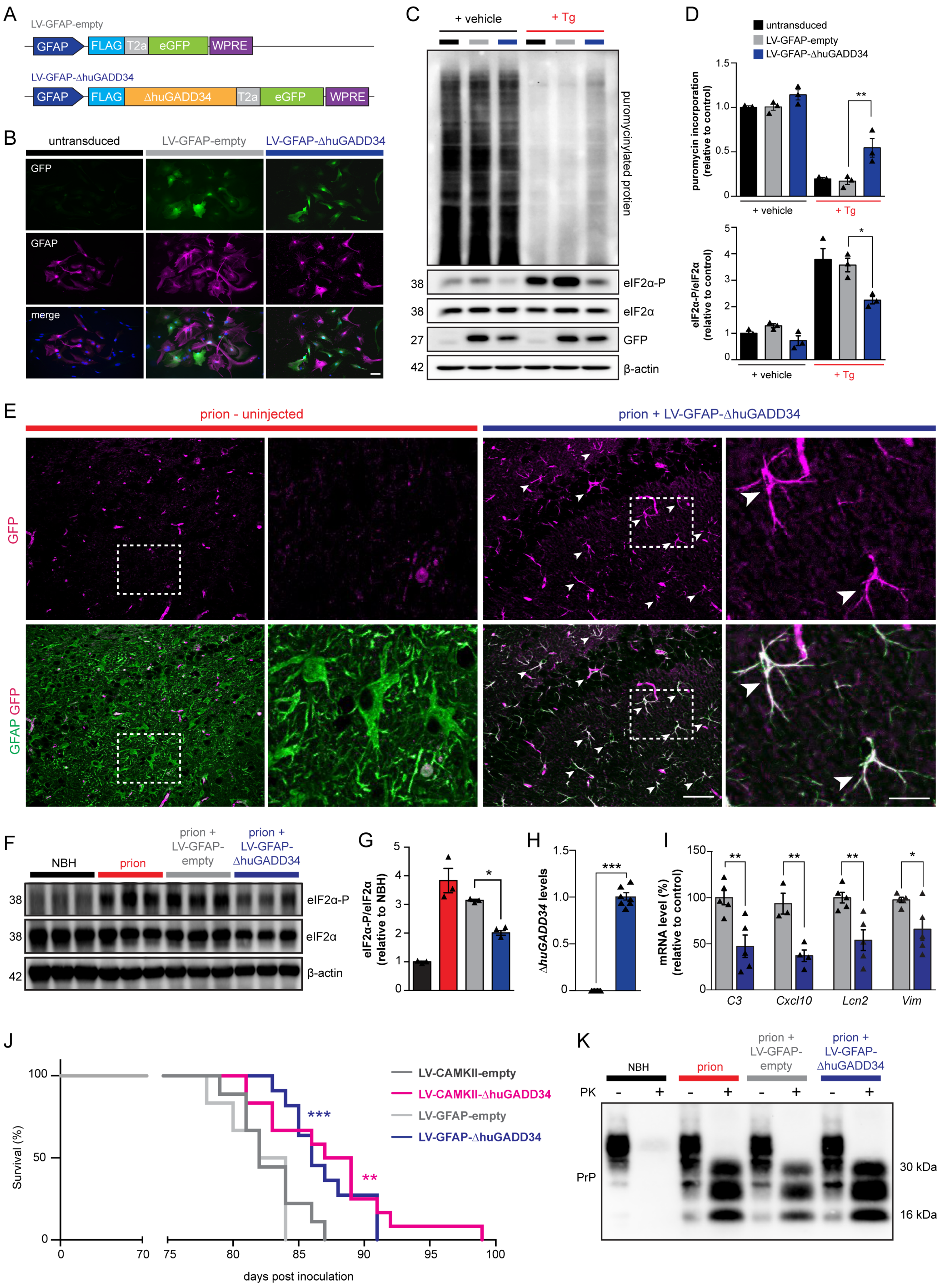

Figure S6 (related to Figures 5 and 6). LV-GFAP-ΔhuGADD34 reduces eIF2α-P levels both *in vitro* and *in vivo*. (A) Schematic showing the GFAP-empty and GFAP-ΔhuGADD34 lentiviral constructs. (**B**) GFP staining in astrocytes transduced with either LV-GFAP-empty or LV-GFAP-ΔhuGADD34 for 7 days. Scale bar, 50 μm. (**C**) Primary astrocytes, transduced with LV-GFAP-empty or LV-GFAP-ΔhuGADD34 for 7 days, were treated with 300 nM Tg for 2 hours. Astrocytes expressing ΔhuGADD34 showed increased protein synthesis rates and lower levels of eIF2α-P on Tg treatment compared to astrocytes expressing GFP alone. Quantification shown in (**D**). Bar graphs show mean ± SEM. **p* <0.05, ***p* <0.01, one-way ANOVA. *n* = 3 biological replicates. (**E**) Representative images of GFP staining in the hippocampus of prion-inoculated mice injected with LV-GFAP-ΔhuGADD34. GFP staining co-localised with GFAP+ astrocytes. The over-expression of astrocytic ΔhuGADD34 substantially reduced the levels of astrocytosis compared to un-injected prion-inoculated mice. Scale bar, 50 μm, zoomed panel, 20 μm. (**F**) Western blot analysis of eIF2α-P in NBH, prion, prion + LV-GFAP-empty and prion + LV-GFAP-ΔhuGADD34 mice. The over-expression of ΔhuGADD34 significantly reduced the levels of eIF2α-P, as quantified in (**G**). Bar graph shows mean ± SEM. **p* <0.05, Student's t-test. *n* = 3 mice per condition. (**H**) Transcript levels of Δ*huGADD34* in prion-inoculated mice injected with either LV-GFAP-empty or LV-GFAP-ΔhuGADD34. Δ*huGADD34* was only detected in LV-GFAP-ΔhuGADD34-treated mice. ****p* <0.001, Student's t-test. *n* = 5 mice per condition. (**I**) The astrocytic expression of ΔhuGADD34 significantly reduced the mRNA levels of the UPR-reactivity markers *C3, Cxcl10, Lcn2* and *Vim* at 10 w.p.i., as analysed by qPCR. **p* <0.05 ***p* <0.01, Student's t-test. *n* = 5 mice per condition. (**J**) Neuronal or astrocytic modulation of PERK-eIF2α both significantly prolonged survival. LV-CAMKII-empty = 9 mice, LV-CAMKII-ΔhuGADD34 = 12 mice, LV-GFAP-empty = 6 mice, LV-GFAP-ΔhuGADD34 = 11 mice. ***p* <0.01, ****p* <0.001, Mantel-Cox test. (**K**) Astrocytic ΔhuGADD34 does not affect the levels of total PrP or proteinase K-resistant PrP^{Sc}.

Table S1 (related to Figure 3). Proteins identified in conditioned media from vehicle-, Tg- and Tg+GSK2606414-treated astrocytes.

			Normalised spectral counts											
			Biological replicate 1			Biological replicate 2			Biological replicate3			Average		
Identified Proteins	Accession Number	Molecular Weight	vehicle	Tg	Tg + GSK	vehicle	Tg	Tg + GSK	vehicle	Tg	Tg + GSK	vehicle	Tg	Tg + GSK
14-3-3 protein epsilon OS=Mus musculus OX=10090 GN=Ywhae PE=1 SV=1	1433E_MOUSE	29 kDa	1.00	1.63	4.88	1.00	0.74	1.63	1.00	0.67	0.81	1.00	1.01	2.44
14-3-3 protein eta OS=Mus musculus OX=10090 GN=Ywhah PE=1 SV=2	1433F_MOUSE	28 kDa	1.00	1.25	5.00	1.00	1.00	2.40	1.00	0.75	0.58	1.00	1.00	2.66
14-3-3 protein gamma OS=Mus musculus OX=10090 GN=Ywhag PE=1 SV=2	1433G_MOUSE	28 kDa	1.00	1.40	3.60	1.00	1.00	1.83	1.00	0.64	0.64	1.00	1.01	2.02
14-3-3 protein theta (Fragment) OS=Mus musculus OX=10090 GN=Ywhaq PE=1 SV=1	F6VW30_MOUSE (+3)	34 kDa	1.00	1.50	6.00	1.00	0.70	1.80	1.00	0.75	0.69	1.00	0.98	2.83
14-3-3 protein zeta/delta OS=Mus musculus OX=10090 GN=Ywhaz PE=1 SV=1	1433Z_MOUSE	28 kDa	1.00	1.50	3.38	1.00	0.64	1.50	1.00	0.79	0.58	1.00	0.98	1.82
6-phosphogluconate dehydrogenase, decarboxylating OS=Mus musculus OX=10090 GN=Pgd PE=1 SV=3	6PGD_MOUSE	53 kDa	-	3.00	12.00	1.00	1.50	3.00	1.00	1.67	1.67	1.00	2.06	5.56
Alpha-2-macroglobulin-P OS=Mus musculus OX=10090 GN=A2m PE=2 SV=2	A2MG_MOUSE	164 kDa	1.00	0.16	0.47	1.00	0.93	0.97	1.00	0.61	0.61	1.00	0.57	0.69
Alpha-enolase OS=Mus musculus OX=10090 GN=Eno1 PE=1 SV=3	ENOA_MOUSE	47 kDa	1.00	1.57	3.86	1.00	0.75	1.38	1.00	0.95	0.77	1.00	1.09	2.00
Amyloid-beta A4 protein OS=Mus musculus OX=10090 GN=App PE=1 SV=1	A0A2I3BPT1_MOUSE (+3)	83 kDa	1.00	1.00	1.00	1.00	0.59	0.94	1.00	0.83	1.33	1.00	0.81	1.09
Annexin A2 OS=Mus musculus OX=10090 GN=Anxa2 PE=1 SV=2	ANXA2_MOUSE	39 kDa	1.00	1.75	2.75	1.00	1.00	1.15	1.00	0.82	0.73	1.00	1.19	1.54
Annexin A3 OS=Mus musculus OX=10090 GN=Anxa3 PE=1 SV=4	ANXA3_MOUSE	36 kDa	1.00	3.00	6.50	1.00	0.67	1.22	1.00	0.94	0.69	1.00	1.53	2.80
Annexin A5 OS=Mus musculus OX=10090 GN=Anxa5 PE=1 SV=1	ANXA5_MOUSE	36 kDa	1.00	6.00	19.00	1.00	0.67	1.17	1.00	0.65	0.59	1.00	2.44	6.92
Apolipoprotein E OS=Mus musculus OX=10090 GN=Apoe PE=1 SV=2	APOE_MOUSE	36 kDa	1.00	1.00	3.25	1.00	0.14	0.93	1.00	0.53	0.53	1.00	0.56	1.57
Arginine-rich, mutated in early stage tumors, isoform CRA_b OS=Mus musculus OX=10090 GN=Manf PE=1 SV=1	Q3TMX5_MOUSE (+1)	20 kDa	-	5.00	2.00	-	3.00	3.00	-	3.00	1.00	-	3.67	2.00
Basement membrane-specific heparan sulfate proteoglycan core protein OS=Mus musculus OX=10090 GN=Hspg2 PE=1 SV=1	B1B0C7_MOUSE (+1)	469 kDa	1.00	0.12	0.94	1.00	0.64	0.93	1.00	0.74	0.74	1.00	0.50	0.87
Beta-actin-like protein 2 OS=Mus musculus OX=10090 GN=Actbl2 PE=1 SV=1	ACTBL_MOUSE	42 kDa	1.00	1.20	4.20	1.00	0.75	0.80	1.00	0.83	0.92	1.00	0.93	1.97
Biglycan OS=Mus musculus OX=10090 GN=Bgn PE=1 SV=1	PGS1_MOUSE	42 kDa	1.00	0.29	0.57	1.00	0.84	1.05	1.00	0.56	0.44	1.00	0.56	0.69
Brain acid soluble protein 1 OS=Mus musculus OX=10090 GN=Basp1 PE=1 SV=3	BASP1_MOUSE	22 kDa	-	3.00	4.00	1.00	0.90	1.10	1.00	1.00	1.75	1.00	1.63	2.28
Caldesmon 1 OS=Mus musculus OX=10090 GN=Cald1 PE=1 SV=1	E9Q0M9_MOUSE	62 kDa	1.00	2.50	6.50	1.00	0.44	1.11	1.00	1.67	3.00	1.00	1.54	3.54
Calreticulin OS=Mus musculus OX=10090 GN=Calr PE=1 SV=1	CALR_MOUSE	48 kDa	1.00	3.25	4.00	1.00	1.13	1.13	1.00	1.20	0.93	1.00	1.86	2.02
Calumenin OS=Mus musculus OX=10090 GN=Calu PE=1 SV=1	CALU_MOUSE	37 kDa	-	6.00	8.00	1.00	0.56	0.56	1.00	1.17	1.17	1.00	2.57	3.24
Carboxypeptidase E OS=Mus musculus OX=10090 GN=Cpe PE=1 SV=2	CBPE_MOUSE	53 kDa	1.00	0.60	1.70	1.00	0.48	0.77	1.00	0.58	0.84	1.00	0.55	1.11
Cathepsin B OS=Mus musculus OX=10090 GN=Ctsb PE=1 SV=2	CATB_MOUSE	37 kDa	1.00	1.13	2.00	1.00	0.47	0.93	1.00	0.65	0.59	1.00	0.75	1.17
Cathepsin Z OS=Mus musculus OX=10090 GN=Ctsz PE=1 SV=1	CATZ_MOUSE	34 kDa	1.00	1.50	2.00	1.00	0.67	1.00	1.00	1.00	1.50	1.00	1.06	1.50
Cluster of Isoform 2 of Tropomyosin beta chain OS=Mus musculus OX=10090 GN=Tpm2 (sp|P58774-2|TPM2_MOUSE)	sp|P58774-2|TPM2_MOUSE [2]	33 kDa	1.00	1.86	3.00	1.00	0.65	0.76	1.00	0.62	0.71	1.00	1.04	1.49
Cofilin-1 OS=Mus musculus OX=10090 GN=Cfl1 PE=1 SV=3	COF1_MOUSE	19 kDa	1.00	7.00	15.00	1.00	3.00	6.50	1.00	0.75	1.00	1.00	3.58	7.50
Collagen alpha-1(I) chain OS=Mus musculus OX=10090 GN=Col1a1 PE=1 SV=4	sp|P11087|CO1A1_MOUSE	138 kDa	1.00	0.67	1.00	1.00	0.65	0.86	1.00	0.61	0.58	1.00	0.64	0.81
Collagen alpha-1(IV) chain OS=Mus musculus OX=10090 GN=Col4a1 PE=1 SV=4	CO4A1_MOUSE	161 kDa	1.00	0.80	1.60	1.00	0.80	1.00	1.00	1.25	1.00	1.00	0.95	1.20
Collagen alpha-1(V) chain OS=Mus musculus OX=10090 GN=Col5a1 PE=1 SV=2	CO5A1_MOUSE	184 kDa	1.00	1.00	1.33	1.00	0.33	0.67	1.00	0.67	0.67	1.00	0.67	0.89
Collagen alpha-2(I) chain OS=Mus musculus OX=10090 GN=Col1a2 PE=1 SV=2	CO1A2_MOUSE	130 kDa	1.00	0.39	0.72	1.00	0.66	1.00	1.00	1.00	1.00	1.00	0.68	0.91
Collagen alpha-2(IV) chain OS=Mus musculus OX=10090 GN=Col4a2 PE=1 SV=4	CO4A2_MOUSE	167 kDa	1.00	0.18	0.91	1.00	0.31	0.77	1.00	1.25	1.00	1.00	0.58	0.89
Collagen alpha-2(V) chain OS=Mus musculus OX=10090 GN=Col5a2 PE=1 SV=1	CO5A2_MOUSE	145 kDa	1.00	0.67	1.29	1.00	0.62	1.09	1.00	1.00	1.00	1.00	0.76	1.12
Complement C3 OS=Mus musculus OX=10090 GN=C3 PE=1 SV=3	sp|P01027|CO3_MOUSE	186 kDa	1.00	0.00	3.00	1.00	0.45	0.70	1.00	2.40	1.00	1.00	0.95	1.57
Creatine kinase B-type OS=Mus musculus OX=10090 GN=Ckb PE=1 SV=1	KCRB_MOUSE	43 kDa	1.00	2.75	7.50	1.00	0.60	1.00	1.00	1.00	1.04	1.00	1.45	3.18
Cystatin-C OS=Mus musculus OX=10090 GN=Cst3 PE=1 SV=2	CYTC_MOUSE	16 kDa	1.00	0.75	2.00	1.00	0.85	0.92	1.00	1.20	1.20	1.00	0.93	1.37
EGF-containing fibulin-like extracellular matrix protein 1 OS=Mus musculus OX=10090 GN=Efemp1 PE=1 SV=1	FBLN3_MOUSE	55 kDa	1.00	0.00	0.67	1.00	0.00	0.40	1.00	0.25	0.75	1.00	0.08	0.61
EGF-containing fibulin-like extracellular matrix protein 2 OS=Mus musculus OX=10090 GN=Efemp2 PE=1 SV=1	FBLN4_MOUSE (+1)	49 kDa	1.00	0.00	1.00	1.00	0.54	0.69	1.00	0.60	0.80	1.00	0.38	0.83
Elongation factor 1-alpha 1 OS=Mus musculus OX=10090 GN=Eef1a1 PE=1 SV=3	EF1A1_MOUSE	50 kDa	1.00	1.00	2.40	1.00	1.00	1.20	1.00	0.83	0.75	1.00	0.94	1.45
Endoplasmic reticulum chaperone BiP OS=Mus musculus OX=10090 GN=Hspa5 PE=1 SV=3	BIP_MOUSE	72 kDa	1.00	2.07	2.43	1.00	2.05	1.48	1.00	1.38	1.12	1.00	1.83	1.67
Endoplasmin OS=Mus musculus OX=10090 GN=Hsp90b1 PE=1 SV=2	ENPL_MOUSE	92 kDa	1.00	5.75	5.00	1.00	1.89	1.37	1.00	1.68	1.11	1.00	3.11	2.49
Extracellular matrix protein 1 OS=Mus musculus OX=10090 GN=Ecm1 PE=1 SV=2	sp|Q61508|ECM1_MOUSE	63 kDa	1.00	0.40	1.20	1.00	0.25	0.50	1.00	0.50	0.67	1.00	0.38	0.79
Ezrin OS=Mus musculus OX=10090 GN=Ezr PE=1 SV=3	EZRI_MOUSE	69 kDa	1.00	1.20	2.40	1.00	0.43	0.79	1.00	0.86	1.00	1.00	0.83	1.40
Farnesyl pyrophosphate synthase OS=Mus musculus OX=10090 GN=Fdps PE=1 SV=1	FPPS_MOUSE	41 kDa	1.00	4.00	8.00	1.00	0.43	1.29	1.00	0.71	0.71	1.00	1.71	3.33
Fibromodulin OS=Mus musculus OX=10090 GN=Fmod PE=2 SV=1	FMOD_MOUSE	43 kDa	1.00	1.00	2.67	1.00	0.31	0.92	1.00	0.33	0.56	1.00	0.55	1.38
Fibronectin OS=Mus musculus OX=10090 GN=Fn1 PE=1 SV=1	A0A087WR50_MOUSE	263 kDa	1.00	0.45	1.03	1.00	0.70	0.89	1.00	0.85	0.77	1.00	0.67	0.89
Fibulin-5 OS=Mus musculus OX=10090 GN=Fbln5 PE=1 SV=1	A0A1Y7VJW9_MOUSE (+1)	52 kDa	1.00	0.33	1.33	1.00	0.53	0.63	1.00	0.22	0.22	1.00	0.36	0.73
Filamin-B OS=Mus musculus OX=10090 GN=Flnb PE=1 SV=3	FLNB_MOUSE	278 kDa	1.00	0.40	4.20	1.00	0.71	1.05	1.00	0.94	0.89	1.00	0.69	2.05
Filamin, alpha OS=Mus musculus OX=10090 GN=Flna PE=1 SV=1	B7FAU9_MOUSE (+2)	280 kDa	1.00	0.88	3.53	1.00	0.69	1.24	1.00	1.14	1.08	1.00	0.90	1.95
Follistatin-related protein 1 OS=Mus musculus OX=10090 GN=Fstl1 PE=1 SV=2	FSTL1_MOUSE	35 kDa	1.00	1.75	1.75	1.00	0.90	1.05	1.00	0.50	0.67	1.00	1.05	1.15
Fructose-bisphosphate aldolase A OS=Mus musculus OX=10090 GN=Aldoa PE=1 SV=2	ALDOA_MOUSE	39 kDa	1.00	2.50	6.75	1.00	0.68	1.05	1.00	0.75	0.96	1.00	1.31	2.92
Galectin-1 OS=Mus musculus OX=10090 GN=Lgals1 PE=1 SV=3	LEG1_MOUSE	15 kDa	1.00	1.50	3.50	1.00	1.00	1.67	1.00	1.67	1.33	1.00	1.39	2.17
Galectin-3-binding protein OS=Mus musculus OX=10090 GN=Lgals3bp PE=1 SV=1	LG3BP_MOUSE	64 kDa	1.00	0.29	0.71	1.00	0.50	0.50	1.00	0.50	1.00	1.00	0.43	0.74
Glucose-6-phosphate isomerase OS=Mus musculus OX=10090 GN=Gpi PE=1 SV=4	G6PI_MOUSE	63 kDa	1.00	9.00	17.00	1.00	0.60	1.27	1.00	1.31	1.23	1.00	3.64	6.50
Glutathione S-transferase Mu 1 OS=Mus musculus OX=10090 GN=Gstm1 PE=1 SV=2	GSTM1_MOUSE	26 kDa	1.00	0.60	3.60	1.00	0.83	1.17	1.00	1.00	1.09	1.00	0.81	1.95
Glutathione S-transferase Mu 2 OS=Mus musculus OX=10090 GN=Gstm2 PE=1 SV=2	GSTM2_MOUSE	26 kDa	1.00	0.33	4.67	1.00	1.00	1.60	1.00	1.33	1.67	1.00	0.89	2.64
Glypican-4 OS=Mus musculus OX=10090 GN=Gpc4 PE=1 SV=2	GPC4_MOUSE	63 kDa	1.00	0.78	1.67	1.00	0.69	0.96	1.00	0.50	0.70	1.00	0.66	1.11
Growth arrest-specific protein 6 OS=Mus musculus OX=10090 GN=Gas6 PE=2 SV=2	GAS6_MOUSE	75 kDa	1.00	0.00	0.60	1.00	0.09	0.18	1.00	0.00	0.00	1.00	0.03	0.26
Heat shock cognate 71 kDa protein OS=Mus musculus OX=10090 GN=Hspa8 PE=1 SV=1	HSP7C_MOUSE	71 kDa	1.00	0.93	2.36	1.00	0.88	1.21	1.00	0.88	0.97	1.00	0.90	1.51
Heat shock protein HSP 90-alpha OS=Mus musculus OX=10090 GN=Hsp90aa1 PE=1 SV=4	HS90A_MOUSE	85 kDa	1.00	1.00	2.63	1.00	0.45	0.90	1.00	1.09	1.04	1.00	0.85	1.52
Heat shock protein HSP 90-beta OS=Mus musculus OX=10090 GN=Hsp90ab1 PE=1 SV=3	HS90B_MOUSE	83 kDa	1.00	1.40	2.50	1.00	0.47	0.94	1.00	1.00	1.00	1.00	0.96	1.48
Insulin-like growth factor-binding protein 2 OS=Mus musculus OX=10090 GN=Igfbp2 PE=2 SV=2	IBP2_MOUSE	33 kDa	1.00	1.18	1.88	1.00	0.62	0.87	1.00	0.56	0.70	1.00	0.78	1.15
Insulin-like growth factor-binding protein 3 OS=Mus musculus OX=10090 GN=Igfbp3 PE=2 SV=2	IBP3_MOUSE	32 kDa	1.00	0.50	1.00	1.00	0.57	1.29	1.00	0.80	0.80	1.00	0.62	1.03
Insulin-like growth factor-binding protein 5 OS=Mus musculus OX=10090 GN=Igfbp5 PE=1 SV=1	IBP5_MOUSE	30 kDa	1.00	0.50	2.00	1.00	0.44	1.22	1.00	1.00	1.50	1.00	0.65	1.57
Isoform 2 of A-kinase anchor protein 12 OS=Mus musculus OX=10090 GN=Akap12	sp|Q9WTQ5-2|AKA12_MOUSE	170 kDa	1.00	2.20	4.20	1.00	0.63	1.16	1.00	1.00	1.04	1.00	1.28	2.13
Isoform 2 of Calsyntenin-1 OS=Mus musculus OX=10090 GN=Clstn1	sp|Q9EPL2-2|CSTN1_MOUSE	108 kDa	1.00	0.20	0.60	1.00	0.75	0.75	1.00	0.63	0.75	1.00	0.53	0.70
Isoform 2 of Fibulin-2 OS=Mus musculus OX=10090 GN=Fbln2	sp|P37889-2|FBLN2_MOUSE (+1)	126 kDa	1.00	0.50	0.00	1.00	0.47	0.53	1.00	0.29	0.71	1.00	0.42	0.42
Isoform 2 of Gelsolin OS=Mus musculus OX=10090 GN=Gsn	sp|P13020-2|GELS_MOUSE	81 kDa	-	3.00	19.00	1.00	3.00	12.00	1.00	0.78	1.33	1.00	2.26	10.78

Table S1 (related to Figure 3). Proteins identified in conditioned media from vehicle-, Tg- and Tg+GSK2606414-treated astrocytes.

			Normalised spectral counts											
			Biological replicate 1			Biological replicate 2			Biological replicate3			Average		
Identified Proteins	Accession Number	Molecular Weight	vehicle	Tg	Tg + GSK	vehicle	Tg	Tg + GSK	vehicle	Tg	Tg + GSK	vehicle	Tg	Tg + GSK
Isoform 2 of Glial fibrillary acidic protein OS=Mus musculus OX=10090 GN=Gfap	sp|P03995-2|GFAP_MOUSE	49 kDa	1.00	2.00	8.50	1.00	1.33	1.17	1.00	0.67	0.67	1.00	1.33	3.44
Isoform 2 of Glucosidase 2 subunit beta OS=Mus musculus OX=10090 GN=Prkcsh	sp|O08795-2|GLU2B_MOUSE (+1)	60 kDa	1.00	5.00	3.00	1.00	2.00	1.50	1.00	1.80	1.60	1.00	2.93	2.03
Isoform 2 of Prolyl 4-hydroxylase subunit alpha-1 OS=Mus musculus OX=10090 GN=P4ha1	sp|Q60715-2|P4HA1_MOUSE	61 kDa	-	9.00	10.00	1.00	4.67	3.33	1.00	4.33	3.33	1.00	6.00	5.56
Isoform 2 of Tropomyosin alpha-1 chain OS=Mus musculus OX=10090 GN=Tpm1	sp|P58771-2|TPM1_MOUSE	33 kDa	1.00	1.40	3.00	1.00	0.70	0.87	1.00	0.78	0.73	1.00	0.96	1.53
Isoform 2 of Tropomyosin alpha-3 chain OS=Mus musculus OX=10090 GN=Tpm3	sp|P21107-2|TPM3_MOUSE	29 kDa	1.00	1.78	3.44	1.00	0.72	0.89	1.00	0.86	0.90	1.00	1.12	1.75
Isoform M1 of Pyruvate kinase PKM OS=Mus musculus OX=10090 GN=Pkm	sp|P52480-2|KPYM_MOUSE	58 kDa	1.00	0.70	2.90	1.00	0.87	1.27	1.00	1.28	1.28	1.00	0.95	1.81
Isoform Short of 14-3-3 protein beta/alpha OS=Mus musculus OX=10090 GN=Ywhab	sp|Q9CQV8-2|1433B_MOUSE (+1)	28 kDa	1.00	2.25	6.00	1.00	0.75	1.50	1.00	0.69	0.56	1.00	1.23	2.69
L-lactate dehydrogenase OS=Mus musculus OX=10090 GN=Ldha PE=1 SV=1	A0A1B0GSX0_MOUSE (+2)	40 kDa	1.00	1.25	2.25	1.00	0.47	0.93	1.00	0.82	0.76	1.00	0.85	1.32
Latent-transforming growth factor beta-binding protein 2 OS=Mus musculus OX=10090 GN=Ltbp2 PE=1 SV=1	E9QNQ3_MOUSE	196 kDa	1.00	0.40	0.80	1.00	0.57	0.86	1.00	0.67	0.33	1.00	0.55	0.66
LIM and SH3 domain protein 1 OS=Mus musculus OX=10090 GN=Lasp1 PE=1 SV=1	LASP1_MOUSE	30 kDa	-	3.00	4.00	1.00	0.33	1.33	1.00	1.00	1.67	1.00	1.44	2.33
Low density lipoprotein receptor-related protein 1 OS=Mus musculus OX=10090 GN=Lrp1 PE=1 SV=1	A0A0R4J0I9_MOUSE (+1)	505 kDa	1.00	0.18	0.76	1.00	0.58	1.12	1.00	0.74	0.74	1.00	0.50	0.87
Low-density lipoprotein receptor-related protein 2 OS=Mus musculus OX=10090 GN=Lrp2 PE=1 SV=1	LRP2_MOUSE	519 kDa	1.00	0.00	0.75	1.00	0.82	1.47	1.00	1.08	0.83	1.00	0.64	1.02
Macrophage colony-stimulating factor 1 OS=Mus musculus OX=10090 GN=Csf1 PE=1 SV=2	sp|P07141|CSF1_MOUSE	61 kDa	1.00	1.00	1.00	1.00	0.83	1.00	1.00	0.67	1.00	1.00	0.83	1.00
Malate dehydrogenase, cytoplasmic OS=Mus musculus OX=10090 GN=Mdh1 PE=1 SV=3	MDHC_MOUSE	37 kDa	1.00	3.00	12.00	1.00	0.50	1.10	1.00	1.14	1.14	1.00	1.55	4.75
Malate dehydrogenase, mitochondrial OS=Mus musculus OX=10090 GN=Mdh2 PE=1 SV=3	MDHM_MOUSE	36 kDa	1.00	2.50	6.50	1.00	0.67	0.83	1.00	1.11	0.89	1.00	1.43	2.74
Metalloproteinase inhibitor 2 OS=Mus musculus OX=10090 GN=Timp2 PE=1 SV=1	Q6PI17_MOUSE (+1)	24 kDa	1.00	0.67	1.33	1.00	0.56	0.67	1.00	0.33	0.42	1.00	0.52	0.81
Moesin OS=Mus musculus OX=10090 GN=Msn PE=1 SV=3	MOES_MOUSE	68 kDa	1.00	1.33	4.00	1.00	0.44	1.00	1.00	0.87	1.00	1.00	0.88	2.00
Myosin-9 OS=Mus musculus OX=10090 GN=Myh9 PE=1 SV=4	MYH9_MOUSE	226 kDa	1.00	5.00	38.00	1.00	0.83	1.24	1.00	1.24	1.12	1.00	2.36	13.45
N(G),N(G)-dimethylarginine dimethylaminohydrolase 1 OS=Mus musculus OX=10090 GN=Ddah1 PE=1 SV=3	DDAH1_MOUSE	31 kDa	-	1.00	1.75	1.00	1.00	1.00	1.00	1.20	0.80	1.00	2.07	2.93
Nestin OS=Mus musculus OX=10090 GN=Nes PE=1 SV=1	sp|Q6P5H2|NEST_MOUSE	207 kDa	1.00	1.00	3.40	1.00	0.17	0.61	1.00	1.11	1.04	1.00	0.76	1.68
Neurocan core protein OS=Mus musculus OX=10090 GN=Ncan PE=1 SV=1	A0A0R4IZX5_MOUSE (+1)	137 kDa	1.00	0.60	1.60	1.00	1.00	1.00	1.00	0.50	1.00	1.00	0.70	1.20
Nidogen-2 OS=Mus musculus OX=10090 GN=Nid2 PE=1 SV=2	NID2_MOUSE	154 kDa	1.00	0.20	1.20	1.00	0.47	0.53	1.00	0.63	0.63	1.00	0.43	0.78
Nucleobindin-1 OS=Mus musculus OX=10090 GN=Nucb1 PE=1 SV=2	NUCB1_MOUSE	53 kDa	1.00	3.25	2.75	1.00	0.75	0.94	1.00	0.58	0.75	1.00	1.53	1.48
Nucleoside diphosphate kinase OS=Mus musculus OX=10090 GN=Gm20390 PE=3 SV=1	E9PZF0_MOUSE	30 kDa	1.00	1.33	3.67	1.00	0.86	1.14	1.00	1.40	1.00	1.00	1.20	1.94
Peptidyl-prolyl cis-trans isomerase A OS=Mus musculus OX=10090 GN=Ppia PE=1 SV=2	PPIA_MOUSE	18 kDa	1.00	1.50	4.00	1.00	0.78	1.44	1.00	1.83	1.83	1.00	1.37	2.43
Peroxiredoxin-1 OS=Mus musculus OX=10090 GN=Prdx1 PE=1 SV=1	PRDX1_MOUSE	22 kDa	1.00	1.00	2.50	1.00	1.00	1.56	1.00	1.14	1.00	1.00	1.05	1.69
Peroxiredoxin-2 OS=Mus musculus OX=10090 GN=Prdx2 PE=1 SV=3	PRDX2_MOUSE	22 kDa	1.00	1.00	2.33	1.00	1.00	1.60	1.00	1.25	1.00	1.00	1.08	1.64
Phosphoglycerate kinase 1 OS=Mus musculus OX=10090 GN=Pgk1 PE=1 SV=4	-	45 kDa	-	5.00	14.00	1.00	1.40	2.60	1.00	1.33	1.33	1.00	2.58	5.98
Pigment epithelium-derived factor OS=Mus musculus OX=10090 GN=Serpinf1 PE=1 SV=2	PEDF_MOUSE	46 kDa	1.00	0.86	1.57	1.00	0.47	0.80	1.00	0.42	0.58	1.00	0.58	0.98
Plasminogen activator inhibitor 1 OS=Mus musculus OX=10090 GN=Serpine1 PE=1 SV=1	G5E899_MOUSE	45 kDa	1.00	0.25	2.75	1.00	0.44	0.89	1.00	0.64	0.71	1.00	0.45	1.45
Prosaposin OS=Mus musculus OX=10090 GN=Psap PE=1 SV=1	E9PZ00_MOUSE (+4)	61 kDa	1.00	0.71	2.86	1.00	0.53	1.13	1.00	0.58	0.83	1.00	0.61	1.61
Proteasome subunit alpha type-1 OS=Mus musculus OX=10090 GN=Psma1 PE=1 SV=1	PSA1_MOUSE	30 kDa	1.00	3.00	10.00	1.00	0.67	0.83	1.00	0.86	0.71	1.00	1.51	3.85
Proteasome subunit alpha type-6 OS=Mus musculus OX=10090 GN=Psma6 PE=1 SV=1	PSA6_MOUSE	27 kDa	1.00	3.00	6.00	1.00	0.67	1.00	1.00	1.50	1.25	1.00	1.72	2.75
Proteasome subunit alpha type-7 OS=Mus musculus OX=10090 GN=Psma7 PE=1 SV=1	PSA7_MOUSE	28 kDa	1.00	1.50	4.00	1.00	1.00	0.83	1.00	0.78	0.89	1.00	1.09	1.91
Protein disulfide-isomerase A3 OS=Mus musculus OX=10090 GN=Pdia3 PE=1 SV=2	PDIA3_MOUSE	57 kDa	1.00	3.33	5.17	1.00	1.32	1.18	1.00	1.30	0.96	1.00	1.99	2.44
Protein disulfide-isomerase A4 OS=Mus musculus OX=10090 GN=Pdia4 PE=1 SV=1	A0A0R4J0Z1_MOUSE (+1)	72 kDa	1.00	7.00	14.00	1.00	1.40	1.60	1.00	2.13	1.13	1.00	3.51	5.58
Protein disulfide-isomerase A6 OS=Mus musculus OX=10090 GN=Pdia6 PE=1 SV=1	Q3TML0_MOUSE	49 kDa	1.00	7.00	9.00	1.00	1.67	1.67	1.00	1.80	1.60	1.00	3.49	4.09
Protein disulfide-isomerase OS=Mus musculus OX=10090 GN=P4hb PE=1 SV=2	PDIA1_MOUSE	57 kDa	1.00	1.40	1.40	1.00	1.42	1.47	1.00	1.00	0.81	1.00	1.27	1.23
Purine nucleoside phosphorylase OS=Mus musculus OX=10090 GN=Pnp PE=1 SV=2	PNPH_MOUSE (+1)	32 kDa	-	5.00	14.00	1.00	0.00	0.88	1.00	1.29	1.29	1.00	2.10	5.39
Rab GDP dissociation inhibitor alpha OS=Mus musculus OX=10090 GN=Gdi1 PE=1 SV=3	GDIA_MOUSE	51 kDa	1.00	3.00	10.00	1.00	0.56	1.67	1.00	1.14	1.43	1.00	1.57	4.37
Rab GDP dissociation inhibitor beta OS=Mus musculus OX=10090 GN=Gdi2 PE=1 SV=1	sp|Q61598|GDIB_MOUSE	51 kDa	1.00	2.50	10.00	1.00	0.67	1.20	1.00	1.50	1.58	1.00	1.56	4.26
Radixin OS=Mus musculus OX=10090 GN=Rdx PE=1 SV=3	RADI_MOUSE	69 kDa	1.00	1.25	3.25	1.00	0.44	1.11	1.00	1.09	1.09	1.00	0.93	1.82
Receptor-type tyrosine-protein phosphatase zeta OS=Mus musculus OX=10090 GN=Ptprz1 PE=1 SV=1	PRPTZ_MOUSE	254 kDa	1.00	0.57	1.00	1.00	0.83	1.06	1.00	0.71	0.57	1.00	0.71	0.88
Serine/threonine-protein phosphatase 2A 65 kDa regulatory subunit A alpha isoform OS=Mus musculus OX=10090 GN=Ppp2r1a PE=1 SV=3	2AAA_MOUSE	65 kDa	-	3.00	4.00	1.00	0.67	2.00	1.00	1.00	1.40	1.00	1.56	2.47
SPARC OS=Mus musculus OX=10090 GN=Sparc PE=1 SV=1	A0A1L1SSH9_MOUSE (+2)	38 kDa	1.00	1.05	1.52	1.00	0.71	0.71	1.00	0.57	0.48	1.00	0.77	0.90
SPARC-like protein 1 OS=Mus musculus OX=10090 GN=Sparcl1 PE=1 SV=3	SPRL1_MOUSE	72 kDa	1.00	0.00	0.33	1.00	0.00	0.18	1.00	1.00	0.67	1.00	0.33	0.39
Tenascin OS=Mus musculus OX=10090 GN=Tnc PE=1 SV=1	sp|Q80YX1|TENA_MOUSE	232 kDa	1.00	0.54	1.46	1.00	1.04	0.92	1.00	0.84	0.80	1.00	0.81	1.06
Thioredoxin domain-containing protein 5 OS=Mus musculus OX=10090 GN=Txndc5 PE=1 SV=1	E9PXX7_MOUSE (+1)	39 kDa	1.00	7.00	13.00	1.00	1.86	1.86	1.00	1.33	1.00	1.00	3.40	5.29
Transgelin OS=Mus musculus OX=10090 GN=Tagln PE=1 SV=3	TAGL_MOUSE	23 kDa	1.00	2.00	4.00	1.00	0.46	1.00	1.00	0.87	0.73	1.00	1.11	1.91
Transitional endoplasmic reticulum ATPase OS=Mus musculus OX=10090 GN=Vcp PE=1 SV=4	TERA_MOUSE	89 kDa	1.00	1.13	3.25	1.00	1.00	1.63	1.00	1.09	1.18	1.00	1.07	2.02
Transketolase OS=Mus musculus OX=10090 GN=Tkt PE=1 SV=1	TKT_MOUSE	68 kDa	1.00	2.33	7.67	1.00	0.88	1.25	1.00	1.06	1.00	1.00	1.42	3.31
Triosephosphate isomerase OS=Mus musculus OX=10090 GN=Tpi1 PE=1 SV=4	TPIS_MOUSE	32 kDa	-	4.00	13.00	1.00	1.00	1.75	1.00	1.75	1.25	1.00	2.25	5.33
Tropomyosin alpha-4 chain OS=Mus musculus OX=10090 GN=Tpm4 PE=1 SV=3	TPM4_MOUSE	28 kDa	1.00	2.60	4.60	1.00	0.83	0.92	1.00	0.60	0.87	1.00	1.34	2.13
Tubulin beta-2A chain OS=Mus musculus OX=10090 GN=Tubb2a PE=1 SV=1	TBB2A_MOUSE	50 kDa	1.00	1.20	2.60	1.00	0.59	0.88	1.00	1.24	1.18	1.00	1.01	1.55
Tubulin beta-4B chain OS=Mus musculus OX=10090 GN=Tubb4b PE=1 SV=1	TBB4B_MOUSE	50 kDa	1.00	1.00	2.50	1.00	0.60	0.80	1.00	1.46	1.46	1.00	1.02	1.59
Tubulin beta-5 chain OS=Mus musculus OX=10090 GN=Tubb5 PE=1 SV=1	TBB5_MOUSE	50 kDa	1.00	1.00	2.83	1.00	0.69	1.00	1.00	1.12	1.12	1.00	0.94	1.65
Vascular cell adhesion protein 1 OS=Mus musculus OX=10090 GN=Vcam1 PE=1 SV=1	Q3UPN1_MOUSE (+1)	81 kDa	1.00	0.67	0.67	1.00	1.00	1.50	1.00	1.33	1.33	1.00	1.00	1.17
Vinculin OS=Mus musculus OX=10090 GN=Vcl PE=1 SV=4	VINC_MOUSE	117 kDa	1.00	0.50	8.50	1.00	1.00	1.68	1.00	1.28	1.24	1.00	0.93	3.81
Vitamin K-dependent protein S OS=Mus musculus OX=10090 GN=Pros1 PE=2 SV=1	PROS_MOUSE	75 kDa	1.00	0.00	0.75	1.00	0.38	0.50	1.00	0.29	0.43	1.00	0.22	0.56
WD repeat-containing protein 1 OS=Mus musculus OX=10090 GN=Wdr1 PE=1 SV=3	WDR1_MOUSE	66 kDa	1.00	0.75	3.00	1.00	0.59	1.12	1.00	1.13	1.27	1.00	0.82	1.79

Table S1 (related to Figure 3). Proteins identified in conditioned media from vehicle-, Tg- and Tg+GSK2606414-treated astrocytes. Astrocyte conditioned media was concentrated and analysed by LC/MS. A protein was identified if it received ≥ 99% confidence with ≥3 unique peptides at 95% confidence in the vehicle- or Tg-treated condition. Only proteins meeting this criterion across all 3 biological replicates were included. Spectral counts were normalised to the vehicle-treated condition; normalised spectral counts from 3 biological replicates are shown.

Table S2 (related to STAR Methods). qPCR primer sequences used in this study.

Primer name	Sequence
Cxcl10 F	ATGACGGGCCAGTGAGAATG
Cxcl10 R	TCGTGGCAATGATCTCAACAC
Lcn2 F	CCACCACGGACTACAACCAG
Lcn2 R	TCCTTGGTTCTTCCATACAGGG
Serpina3n F	CCTGGAGGATGTCCTTTCAA
Serpina3n R	TTATCAGGAAAGGCCGATTG
Vim F	GCAGTATGAAAGCGTGGCT
Vim R	CTCCAGGGACTCGTTAGTGC
C3 F	AGCTTCAGGGTCCCAGCTAC
C3 R	GCTGGAATCTTGATGGAGACGC
ΔhuGADD34 F	GGACCCTGAGACTCCCCTAAA
ΔhuAGDD34 R	AGGAAATGGACAGTGACCTTCT
